# Probabilistic linguistic fuzzy cognitive maps: applications to the critical factors affecting the health of rural older adults

**DOI:** 10.1186/s12911-022-02028-9

**Published:** 2022-11-17

**Authors:** Jian Wu, Yucheng Chen, Zengwen Wang, Guoheng Hu, Chen Chen

**Affiliations:** 1 The Center for Social Security Studies, Wuhan University, Wuhan, 430072 China; 2 School of Political Science and Law, University of Jinan, Shandong, 250022 China

**Keywords:** The health of rural older adults, Probabilistic linguistic fuzzy cognitive maps, Cause–effect analysis, Similarity measure

## Abstract

**Background:**

Achieving healthy ageing has become the only way for China to alleviate the pressure of ageing, especially in rural areas. However, the factors affecting the health of rural older adults are numerous and complex. It is important to identify the critical factors that affecting the health of older adults in rural areas and provide decision-making support for targeted health interventions.

**Methods:**

To overcome some limitations of existing works, an extended probabilistic linguistic fuzzy cognitive map model is proposed in this paper as a useful tool for modeling the cause-effect relationship between factors. The proposed model integrates the advantages of probabilistic linguistic term sets and fuzzy cognitive maps. In the end, to rank and identify the critical factors affecting the health, a novel similarity measure based on Euclidean distance and Z-mapping function is proposed.

**Results:**

The proposed model can effectively deal with the uncertainty of experts and reflect different opinions of groups well. In terms of representing uncertainty and ambiguity, the proposed method outperforms other models in modeling complex systems. In the real-world case analysis, we find that *education* is the most important factor affecting the health of rural older adults, followed by *previous occupational experiences*, *psychology*, and *physical exercise*, among other things. *Intergenerational relationship* has become another important factor affecting the health of rural older adults in China as the development of Chinese society.

**Conclusions:**

From a macro perspective, *social economic status*, *living environment*, *lifestyle*, and *health management*, are the variables that have the greatest impact on the health of rural older adults. As a result, providing more precise health interventions with the characteristics of factors influencing health is a crucial guarantee for preserving and improving the health of rural older adults in China.

**Supplementary Information:**

The online version contains supplementary material available at 10.1186/s12911-022-02028-9.

## Introduction

In the context of aging, the health problems of older adults have become a global issue. China is rapidly ageing at the moment. According to National Bureau of Statistics data, China’s population of people aged 60 and up increased from 90 million in 2001 to 267 million in 2021.^1^ The physiological function of older adults gradually declines as they age, and their morbidity is also higher than that of other age groups. According to *Analysis Report of National Health Services Survey*, the prevalence of chronic diseases in older adults is 71%,^2^ while the proportion of deaths caused by chronic diseases has reached 86%^3^ in China. Chronic diseases not only reduce the quality of life of older adults, but also increase medical expenses and the cost of care, imposing a significant socioeconomic burden. Rural older adults are at a disadvantage in term of economic conditions, community service resources and the health knowledge when compared to urban older adults. Against this backdrop, it is important to pay more attention to the health of rural older adults. To provide precise decision-making supports for maintaining and improving their health, it is important to identify the critical factors affecting the health of rural older adults.

And it is common knowledge that there are many different and intricate elements that impact health. Previous research demonstrated that a variety of factors, such as *natural conditions*, *socioeconomic status*, *lifestyle*, *sickness*, *access to healthcare*, *individual demographic characteristics*, etc. [1–6], have an impact on health. At present, the existing methods for analyzing the factors affecting the health of older adults primarily consider the impact of a single factor or a small number of factors on health, and few consider the interaction between factors or the reverse impact of health on its factors. However, holistic human health systems are intricate and dynamic. At the same time, the factors that affect health are diverse and interdependent. Consequently, it is inevitable that the coupling effects between factors and the interaction effects between factors and health in the cause–effect analysis of health. Therefore, it is more appropriate to consider the analysis of the factors affecting the health of older adults as a complex system.

In this paper, a novel probabilistic linguistic fuzzy cognitive maps (PLFCMs) model, which is combined with probabilistic linguistic term sets (PLTSs) and fuzzy cognitive maps (FCMs), is proposed to simulate the real-world case of factors affecting the health of rural older adults in this paper. It can deal with hesitancy and uncertainty in the evaluation process. In terms of handling uncertainty in human reasoning process, it is more flexibly than traditional FCMs models. And it is also more suitable for human cognitive representation.

The structure of this paper is as follows. A concise overview of two areas of research involved in “Related works” section (i.e., factors affecting the health of rural older adults, and FCMs). Some basic concepts about PLTSs and HFLCMs are introduced in “Preliminary” section. “The construction of PLFCMs and specific decision-making process” section describes the construction of PLFCMs and specific fuzzy decision-making analysis method. The index system is constructed in “Materials” section. In “Results” section, the analytical outcomes, sensitivity analyses, and comparative analyses are presented. Conclusions and policy implications are presented in “Conclusion and policy implication” section.

## Related works

### The factors that affect the health of rural older adults

Currently, a great deal of research is devoted to the three categories of factors on health: *individual lifestyle*, *social factors*, and *medical factors*. However, these factors do not affect health independently; rather, they have a coupling and mutual influence relationship. On the one hand, *individual lifestyle* is typically a mediating variable for social factors affecting health [4, 7]. In terms of *socioeconomic status*, previous works found that *education*, *income* and *social network* can affect health by *individual lifestyle* [4, 8–11]. While with respect to *social environment*, the use of the internet, the participation in social activities, and the community sports infrastructure can all influence individual lifestyle and affect health outcomes [12–14]. Social factors, on the other hand, have a medical impact on health. Previous research has found that the higher one’s socioeconomic status, the higher the quality of medical services and health conditions [15]. At the same time, the availability of medical treatment (visiting hours and prices of medical services) can have an impact on patients’ health by the utilization of medical services [16]. In contrast, the utilization of medical services can alter individual lifestyle and have an impact on their health. People, for example, usually adjust their diet in a healthier direction after learning that they have hypertension [17]. What’s more, while all these three factors (*individual lifestyle*, *social factors*, and *medical factors*) affect health, the direction of influence may differ. Individual lifestyle, for example, includes both healthy (exercise, rest, etc.) and unhealthy (smoking, drinking, etc.) behaviors that may be beneficial or detrimental to health. The same is true for *socioeconomic status*; those with high socioeconomic status have both healthy and unhealthy lifestyles [18]. Besides, excessive use of medical services is harmful to one’s health. As a result, health-related factors are extremely complex, with varying degree of influence and directions on health.

Previous research has examined health-related factors from a variety of perspectives, and these studies serve as important references for this paper. Most of these studies focus on determining which factors have an impact on health, or on the causal relationship between the factors and health, or on the mechanism between them. However, health status is the result of several factors affecting health, and these factors interact in the real-world. Previous studies failed to consider the interrelationship of various factors in the analysis process. Furthermore, while WHO has reported the degree of influence of lifestyle, environment, genetic and medical factors on health, each of these factors contains many small units (for example, lifestyle, which includes diet, exercise, smoking, drinking, and so on), and the impact of these units on health is also uncertain for different groups of people.

To achieve healthy ageing and make precise interventions on the health of rural older adults in China, it is necessary to identify the critical and detailed factors affecting the health of rural older adults. Against this backdrop, a framework of factors affecting health is constructed from four aspects: *the lifestyle of the rural older adults*, *the health management of the rural older adults*, *socio-economic status* and *living environment*.

### Fuzzy cognitive maps

There are several literature on cause analysis, such as HMM [19], based-Bayesian method [20], DEMATEL [21] and so on. However, there are some drawbacks. For example, the based-Bayesian model must be constructed under an assumption that all attributes are mutually independent, and it is sensitive to the input data. And the hesitancy of experts is ignored and the criteria states are required to be linearly interactive in DEMATEL method. FCMs is used in this paper to model the human health system because of their ability to model complex systems with limited data and reduce direct reliance on expert opinions through learning algorithms. Fuzzy cognitive maps (FCMs) is a soft computing method that can be utilized in identifying, describing, and modeling complex systems [22]. The nodes and directed edges in FCMs respectively represent the concepts (such as the characteristics, attributes, variables, states, outputs, qualities, etc.) and their relationship, which can accurately reflect the interaction relationships between different variables. Scholars have paid close attention to build an FCMs model in recent years. Regarding the construction methods of FCMs, there are mainly two methods, one is based on expert-driven approach, the other is based on learning algorithms. The expert-driven methods for establishing FCMs are most based on experts’ knowledge discovery and representation. The based-learning algorithms methods are most based on divide and conquer method [23], Hebbian-based learning [24], particle swarm optimization [25], real-coded genetic algorithm [26] etc. These methods provide good theoretical support for modeling FCMs model.

However, there are numerous sources of uncertainty in the construction process. Various uncertain theories are proposed to deal with the uncertainty and applied in extending the FCMs models. The Fuzzy sets (FSs) theory was proposed by Zadeh [27] as one of most useful methods for representing cognition. In addition, many extensions of FSs, such as intuitionistic fuzzy sets [28], hesitant fuzzy sets [29] etc., are being investigated. Based on the extensions of FSs, some extended FCMs models are constructed, such as intuitionistic fuzzy cognitive maps [30, 31], hesitant fuzzy linguistic cognitive maps (HFLCMs) [32], hesitant fuzzy cognitive maps [33] and so on. Besides, grey system theory (GST), as another method for portraying uncertainty, was proposed by Julong [34] Deng. Salmeron [35] proposed fuzzy gray cognitive maps based on GST theory. In addition, other extensions of FCMs model have also been developed [36, 37].

And, due to the limitations of experts’ experience, knowledge, cognitive capacity, and so on, it may result in inconsistent results when the experts assess in the constructing process for the connection matrix. Meanwhile, with the increasing complexity of the decision-making environment, the above extensions of FCMs models fail to satisfy the need for knowledge inference. In terms of large-scale systems, the workload for the building FCMs model by a single expert would be substantial. So, in the real world, the construction process for connection matrix requires more than one expert to complete. To retain more information in an uncertain environment, the PLTSs is utilized in indicating the value of concepts and the weights of directed edges in this paper, thereby avoiding the complicated subsequent construction process. Furthermore, the steps of divide and conquer in the large-scale system could be avoided. In this paper, a novel of FCMs model based on PLTSs is developed, which can effectively overcome some of the drawbacks of the previously mentioned extensions of FCMs model.

Motivated by the above review, the main contributions of this paper are summarized as below.

1. A novel FCM model called PLFCMs is proposed to analyze the factors affecting the health of rural older adults in China in this paper.
2. A novel similarity measure for PLTSs is proposed to rank and distinct the factors affecting the health of rural older adults. And some properties have been discussed in this paper.
3. The factors affecting the health of rural older adults are viewed for the first time as a complex and holistic system.

## Preliminary

Some basic concepts of PLTSs and HFLCMs are introduced in this section to help readers better understand the theoretical knowledge and mathematical model.

### The concept of probabilistic linguistic term sets

#### Definition 1

[38] Suppose $L = \{ L_{\alpha } |\alpha = - \tau , \ldots , - 1,0,1, \ldots ,\tau \}$ is a linguistic term set (LTs), then the definition of the probabilistic linguistic term set is:

1 $$L(P) = \left\{ {L^{\theta } (p^{\theta } )|L^{\theta } \in L,\,\,p^{\theta } \ge 0,\,\,\theta = 1,2, \ldots ,\# L(P),\sum\limits_{\theta = 1}^{\# L(P)} {p^{\theta } } \le 1} \right\}$$

where $L^{\theta } (p^{\theta } )$ is the linguistic term $L^{\theta }$ associated with the probability $p^{\theta }$, and $\# L(P)$ represents the number of all different linguistic terms in $L(P)$.

#### Definition 2

[38] Suppose a PLTS $L(P)$ with $\sum\limits_{\theta = 1}^{\# L(P)} {p^{\theta } } \le 1$, the associated PLTS $\dot{L}(\dot{P}) = \{ \dot{L}^{\theta } (\dot{P}^{\theta } )\}$ is called as a standard PLTS, where $0 \le \dot{p}^{\theta } { = }p^{\theta } /\sum\nolimits_{\theta = 1}^{{\# \dot{L}(\dot{P})}} {\dot{p}^{\theta } \le 1}$ for all $\theta = 1,2, \ldots ,\# \dot{L}(\dot{P})$. And the score of $\dot{L}(\dot{P})$ is $E\left( {\dot{L}(\dot{P})} \right) = s_{{\overline{\alpha }}} ,\overline{\alpha } = \sum\nolimits_{\theta = 1}^{{\# \dot{L}(\dot{P})}} {L_{1}^{\theta } \dot{P}_{1}^{\theta } }$.

#### Definition 3

[38] Let $L_{1} (P_{1} )$ and $L_{2} (P_{2} )$ be two PLTSs, $L_{1} (P_{1} ) = \{ L_{1}^{\theta } (P_{1}^{\theta } )|\theta = 1,2, \ldots ,\# L_{1} (P_{1} )\}$,$L_{2} (P_{2} ) = \{ L_{2}^{\theta } (P_{2}^{\theta } )|\theta = 1,2, \ldots ,\# L_{2} (P_{2} )\}$, and let $\# L_{1} (P_{1} )$ and ${{\# }}L_{2} (P_{2} )$ be the numbers of LTs in $L_{1} (P_{1} )$ and $L_{2} (P_{2} )$ correspondingly. Supposing $\# L_{1} (P_{1} ) \ge {{\# }}L_{2} (P_{2} )$, in order to make the numbers of LTs in $L_{1} (P_{1} )$ and $L_{2} (P_{2} )$ are same, we can add $\# L_{1} (P_{1} ) - {{\# }}L_{2} (P_{2} )$ LTs to $L_{2} (P_{2} )$, and the corresponding probabilities are zeros.

Based on Definitions 2 and 3, we can get the normalized PLTSs as $L^{N} (P) = \{ L^{N(\theta )} (P^{N(\theta )} )\}$, where $P^{N(\theta )} = {{P^{\theta } } \mathord{\left/ {\vphantom {{P^{\theta } } {\sum\nolimits_{\theta = 1}^{\# L(P)} {P^{\theta } } }}} \right. \kern-\nulldelimiterspace} {\sum\nolimits_{\theta = 1}^{\# L(P)} {P^{\theta } } }}$ for all $\theta = 1,2, \ldots ,\# L(P)$.

#### Definition 4

[39, 40] Let $L_{1} (P_{1} )$ and $L_{2} (P_{2} )$ be two PLTSs, $\lambda$ be a positive real number, $\eta_{1}^{(i)} \in g\left( {L_{1} \left( P \right)} \right)$,$\eta_{2}^{(j)} \in g\left( {L_{2} \left( P \right)} \right)$, where $i = 1,2, \ldots ,\# L_{1} (P)$;$j = 1,2, \ldots ,\# L_{2} (P)$. The $g$ and $g^{ - 1}$ are two equivalent transformation functions. So, the operational laws of PLTSs are shown as follows:

2 $$\begin{aligned} g:\left[ { - \tau ,\tau } \right] \to \left[ {0,1} \right],g\left( {L_{1} \left( P \right)} \right) = & \left\{ {\left( {\frac{{\gamma ^{\theta } + \tau }}{{2\tau }}} \right)\left( p \right)} \right\} \\ = & \left\{ {\left( {\eta ^{\theta } } \right)\left( p \right)} \right\},\eta \in \left[ {0,1} \right] \\ \end{aligned}$$

3 $$\begin{aligned} & g^{{ - 1}} :\left[ {0,1} \right] \to \left[ { - \tau ,\tau } \right],g^{{ - 1}} \left( {g\left( {L_{1} \left( P \right)} \right)} \right) \\ & \quad = \left\{ {s_{{\left( {2\eta - 1} \right)\tau }} \left( p \right)|\eta \in \left[ {0,1} \right]} \right\} = L_{1} \left( P \right) \\ \end{aligned}$$

4 $$\begin{aligned} & L_{1} \left( {P_{1} } \right) \oplus L_{2} \left( {P_{2} } \right) = g^{{ - 1}} \\ \left( {\bigcup\limits_{{\eta _{1}^{{\left( i \right)}} \in g\left( {L_{1} \left( {P_{1} } \right)} \right),\eta _{2}^{{\left( j \right)}} \in g\left( {L_{2} \left( {P_{2} } \right)} \right)}} {\left\{ {\eta _{1}^{{\left( i \right)}} + \eta _{2}^{{\left( j \right)}} - \eta _{1}^{{\left( i \right)}} \eta _{2}^{{\left( j \right)}} \left( {p_{1} p_{2} } \right)} \right\}} } \right) \\ \end{aligned}$$

5 $$L_{1} \left( {P_{1} } \right) \otimes L_{2} \left( {P_{2} } \right) = g^{ - 1} \left( {\bigcup\limits_{{\eta_{1}^{\left( i \right)} \in g\left( {L_{1} \left( {P_{1} } \right)} \right),\eta_{2}^{\left( j \right)} \in g\left( {L_{2} \left( {P_{2} } \right)} \right)}} {\left\{ {\eta_{1}^{\left( i \right)} \eta_{2}^{\left( j \right)} \left( {p_{1} p_{2} } \right)} \right\}} } \right)$$

6 $$\lambda L_{1} \left( P \right) = g^{ - 1} \left( {\bigcup\limits_{{\eta_{1}^{\left( i \right)} g\left( {L_{1} \left( P \right)} \right)}} {\left\{ {1 - \left( {1 - \eta_{1}^{\left( i \right)} } \right)^{\lambda } \left( p \right)} \right\}} } \right)$$

7 $$\left( {L_{1} \left( P \right)} \right)^{\lambda } = g^{ - 1} \left( {\bigcup\limits_{{\eta_{1}^{\left( i \right)} g\left( {L_{1} \left( P \right)} \right)}} {\left\{ {\left( {\eta_{1}^{\left( i \right)} } \right)^{\lambda } \left( p \right)} \right\}} } \right)$$

#### Definition 5

[38] Let $L_{1} (P_{1} )$ and $L_{2} (P_{2} )$ be two PLTSs, the probabilistic linguistic Euclidean distance between $L_{1} (P_{1} )$ and $L_{2} (P_{2} )$ are defined as.

8 $$d\left( {L_{1} \left( P \right),L_{2} \left( P \right)} \right) = \sqrt {\frac{1}{l}\sum\limits_{\theta = 1}^{l} {\left( {L_{1}^{\theta } \left( {p^{\theta } } \right) - L_{2}^{\theta } \left( {p^{\theta } } \right)} \right)^{2} } }$$

where $l = \max \left\{ {\# L_{1} ,\# L_{2} } \right\}$ is the length of $L_{1} (P)$ and $L_{2} (P)$.

#### Definition 6

[41] Let $L_{1} (P_{1} )$ and $L_{2} (P_{2} )$ be two PLTSs, the similarity degree $S$ is defined as below:

9 $$S\left( {L_{1} \left( P \right),L_{2} \left( P \right)} \right) = \frac{{Z\left( {d\left( {L_{1} \left( P \right),L_{2} \left( P \right)} \right)} \right) - Z\left( 1 \right)}}{Z\left( 0 \right) - Z\left( 1 \right)}$$

here $d\left( {L_{1} \left( P \right),L_{2} \left( P \right)} \right)$ is the probabilistic linguistic Euclidean distance between $L_{1} (P_{1} )$ and $L_{2} (P_{2} )$, and $Z:[0,1] \to [0,1][0,1]$ is a mapping function. The usual form of $Z$ mapping function are: (1) $Z(t) = 1 - t$; 2) $Z(t) = \frac{1 - t}{{1 + t}}$; (3) $Z(t) = 1 - te^{t - 1}$; (4) $Z(t) = 1 - t^{2}$.

#### Property 1

Let $L_{1} (P_{1} )$ and $L_{2} (P_{2} )$ be two PLTSs. Assume $S$ is a similarity degree between $L_{1} (P_{1} )$ and $L_{2} (P_{2} )$, then we have:

1. $$0 \le S\left( {L_{1} \left( P \right),L_{2} \left( P \right)} \right) \le 1$$.
2. $$S\left( {L_{1} \left( P \right),L_{2} \left( P \right)} \right) = S\left( {L_{2} \left( P \right),L_{1} \left( P \right)} \right)$$.
3. $S\left( {L_{1} \left( P \right),L_{2} \left( P \right)} \right) = 0$ if and only if $L_{1} \left( P \right) = L_{2}^{c} \left( P \right)$ or $L_{2} \left( P \right) = L_{1}^{c} \left( P \right)$.
4. $S\left( {L_{1} \left( P \right),L_{2} \left( P \right)} \right) = 1$ if and only if $L_{1} \left( P \right) = L_{2} \left( P \right)$.

#### Proof

Without loss of generality, suppose $Z(t) = 1 - t$, and the process of proof is shown as below:

1. Since $d\left( {L_{1} \left( P \right),L_{2} \left( P \right)} \right) \in \left[ {0,\,1} \right]$, then $Z\left( {d\left( {L_{1} \left( P \right),L_{2} \left( P \right)} \right)} \right)$ will be in $\left[ {0,1} \right]$. So, $S_{d} \left( {L_{1} \left( P \right),L_{2} \left( P \right)} \right) = Z\left( {d\left( {L_{1} \left( P \right),L_{2} \left( P \right)} \right)} \right) \in \left[ {0,1} \right]$.
2. Since $d\left( {L_{1} \left( P \right),L_{2} \left( P \right)} \right) = \sqrt {\frac{1}{l}\sum\limits_{\theta = 1}^{l} {\left( {L_{1}^{\theta } \left( {p^{\theta } } \right) - L_{2}^{\theta } \left( {p^{\theta } } \right)} \right)^{2} } } = \sqrt {\frac{1}{l}\sum\limits_{\theta = 1}^{l} {\left( {L_{2}^{\theta } \left( {p^{\theta } } \right) - L_{1}^{\theta } \left( {p^{\theta } } \right)} \right)^{2} } } = d\left( {L_{2} \left( P \right),L_{1} \left( P \right)} \right)$ then we have $S\left( {L_{1} \left( P \right),L_{2} \left( P \right)} \right) = S\left( {L_{2} \left( P \right),L_{1} \left( P \right)} \right)$.
3. When $S\left( {L_{1} \left( P \right),L_{2} \left( P \right)} \right) = 0$, it means $Z\left( {d\left( {L_{1} \left( P \right),L_{2} \left( P \right)} \right)} \right) = Z\left( 1 \right)$. Then $d\left( {L_{1} \left( P \right),L_{2} \left( P \right)} \right)$ equals to 1. So $L_{1} \left( P \right) = L_{2}^{c} \left( P \right)$ or $L_{2} \left( P \right) = L_{1}^{c} \left( P \right)$ must be satisfied. And vice versa.
4. When $S\left( {L_{1} \left( P \right),L_{2} \left( P \right)} \right) = 1$, it means $Z\left( {d\left( {L_{1} \left( P \right),L_{2} \left( P \right)} \right)} \right) = Z\left( 0 \right)$. Then $d\left( {L_{1} \left( P \right),L_{2} \left( P \right)} \right)$ equals to 0. So $L_{1} \left( P \right) = L_{2} \left( P \right)$ must be satisfied. And vice versa.

For other forms of Z mapping functions, the proof is the same as above. So, we finish the proof for this property.

### A simple hesitant fuzzy linguistic cognitive maps model

**Fig. 1:**
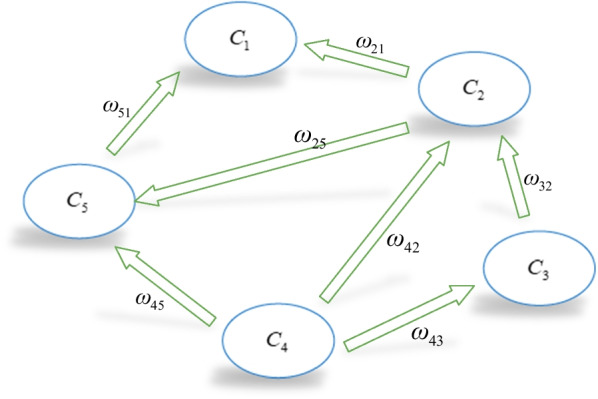
A simple HFLCMs model

FCMs is a useful soft computing tool for modeling complex systems that combines fuzzy logic and neural networks [42], and is made up of nodes and directed edges. The nodes can represent the system’s behavioral characteristics, such as causes, variables, states, and so on. The directed edges not only represent the causal relationships from starting point to ending point but also their weight. Hesitant fuzzy linguistic cognitive maps (HFLCMs), as an important extension of classical FCMs model, is presented by Çoban and Onar [32]. To intuitively demonstrate the model and its principles, a simple HFLCMs model is shown in Fig. 1.

Figure 1 depicts a simple HFLCMs model comprising of five concepts. Generally, concepts represent the states, variables, and conceptual characteristics of the system. The directed edge, which represents the causal relationships between $C_{i}$ and $C_{j}$, is represented by hesitant fuzzy linguistic terms sets (HFLTSs), such as “at least medium”, “greater than low”, “between low and very high” and so on. Then these type of hesitant linguistic expressions and linguistic term in a nature language can be generated using context-free grammar. It can denote the influence degree of one concept on another.

Generally, the weights of edges can be obtained from experts’ knowledge [30, 31], and they always are transformed into trapezoidal fuzzy number for computing. Then, the trapezoidal fuzzy membership functions are transformed within the [− 1, 1] interval by weighted average defuzzification method [32]. Therefore, there is a transition matrix W, which corresponds to the simple HFLCMs model in Fig. 1. Similarly, the concept value $x_{i}$ is a degree corresponding to its physical value, which also is represented in the form of HFLTSs. And concept values are transformed into interval [− 1,1].

$$W = \left( {\begin{array}{*{20}c} 0 & 0 & 0 & {\begin{array}{*{20}c} 0 & 0 \\ \end{array} } \\ {\omega_{21} } & 0 & 0 & {\begin{array}{*{20}c} 0 & {\omega_{25} } \\ \end{array} } \\ 0 & {\omega_{32} } & 0 & {\begin{array}{*{20}c} 0 & 0 \\ \end{array} } \\ {\begin{array}{*{20}c} 0 \\ {\omega_{51} } \\ \end{array} } & {\begin{array}{*{20}c} {\omega_{42} } \\ 0 \\ \end{array} } & {\begin{array}{*{20}c} {\omega_{43} } \\ 0 \\ \end{array} } & {\begin{array}{*{20}c} {\begin{array}{*{20}c} 0 \\ 0 \\ \end{array} } & {\begin{array}{*{20}c} {\omega_{45} } \\ 0 \\ \end{array} } \\ \end{array} } \\ \end{array} } \right)$$

In general, for a HFLCMs model with *n* nodes, to ensure that the node values of each iteration are within the interval value, a threshold function $f(x)$ is required for mapping. And the value of each node at time $t + 1$ can be calculated by the following formula:

10 $$x_{i} \left( {t + 1} \right) = f\left( {x_{i} \left( t \right) + \sum\limits_{j = 1,j \ne i}^{n} {x_{j} \left( t \right) \cdot \omega_{ji} } } \right)$$

here $x_{i} \left( {t + 1} \right)$ denotes the value represented by HFLTSs of concept $C_{i}$ at step $t + 1$;$x_{j} (t)$ implies the value represented by HFLTSs of concept $C_{j}$ at step $t$;$\omega_{ji}$ is the weight that implies the influence degree of concept $C_{j}$ on $C_{i}$;$f(x)$ represents the threshold function. In most cases, threshold functions such as unipolar sigmoid function and the hyperbolic tangent are utilized in HFLCMs model [43].

The initial state of HFLCMs is determined by experts. The system will then end up with three situations as a result of the iteration formula in Eq. (10): (1) it can be stabilized to a fixed point; (2) it can be caught in a limit cycle; (3) it can end up with a chaotic attractor [43]. If the system reaches the situation (1) or (2), the iteration and the reasoning process can be stopped. When the system ends up with situation (3), the system cannot be illustrated and should be rebuilt.

## The construction of PLFCMs and specific decision-making process

When experts give the relationships between factors or between factors and health, they frequently are unable to express their opinions or judgments accurately due to the limitation in their own knowledge and experience or the ambiguity of their opinions. For example, when asked “*how do you feel about your physical condition?*”, experts may hesitate between “*medium*” or “*slightly bad*”. During statistical surveys, experts frequently use only one word to describe the evaluation information, such as “*medium*” or “*slightly bad*”. This is obviously problematic, as it limits the expression and choice of information by experts. Therefore, hesitant fuzzy linguistic terms set is proposed by Rodríguez [44]. Moreover, in the majority of instances, experts do not share the same preference for evaluation value. If the expert prefers “*medium*” or “*slightly bad*” in the scenario presented above, the case can only be expressed using HFLTS {“*medium*” or “*slightly bad*”}. This is also clearly problematic, as it does not reflect the expert’s preference for one evaluation criterion over another. Therefore, HFLTSs also does not meet the demands of decision-makers. To overcome the above situations, Pang et al. [38] proposed probabilistic linguistic term sets for expressing decision-making information. Thus, the above case may be expressed by {*medium* (0.6), *slightly bad* (0.4)}. PLTSs have several benefits, including a more realistic depiction of uncertain forms and the storage of more evaluation information than conventional linguistic terms, etc*.* (See references [45, 46] for further information).

Considering the uncertainty and ambiguity of human cognition in modeling complex system, we introduce PLTSs for expressing the evaluation from experts in this section. Specifically, we construct a novel FCMs model called PLFCMs, which integrates the advantages of PLTSs and FCMs. On this basis, we will identify the most influential factors on the health of rural older adults. The novel model is applied to analyze the causal relationship between the factors influencing health and their effects on the health of rural older adults.

### The construction of PLFCMs and the inference process

To make the PLFCMs method more intuitive, we will explain the specific mathematical construction and the inference procedure in this section.

#### Definition 7

PLFCMs is a four-tuple structure $G = \left( {C,E,X,f} \right)$, where $C = \left\{ {C_{1} ,C_{2} , \ldots ,C_{n} } \right\}$ is a set of nodes. $C_{i} = (L^{i} (p))$ is a probabilistic linguistic element (PLE), where $n$ is the number of nodes. $E:(C_{i} ,C_{j} ) \to \omega_{ij}$ is a mapping, and $\omega_{ij} (p)$ is a PLE, which represents the causal relationship between any two concepts, and $X:V_{i} \to x_{i}$ is a mapping. Here,$x_{i} (t)$ represents the state value of node $V_{i}$ at time $t$.$X\left( t \right) = \left[ {x_{1} \left( t \right),x_{2} \left( t \right), \ldots ,x_{n} \left( t \right)} \right]$ represents the state vector of PLFCMs $G$ at time $t$.$f(x)$ is the threshold function, which ensures that the node values of each iteration are in the interval [0,1].

#### Definition 8

(*Concept node*) The nodes in the PLFCMs are called concept nodes, which can represent entities, actions, behaviors, causes, trends, and indicators in the system, etc. It is denoted as $C_{i} = (L^{i} (p))$, which represents the $i$-th concept node in $G$.

#### Definition 9

(*Weight*) In PLFCMs, for two different concept nodes $C_{i}$ and $C_{j}$ in $C$, if there is a direct causal relationship between $C_{i}$ and $C_{j}$, then a PLE $\omega_{ij} (p)\;(\omega_{ij} (p) \in E)$ is utilized in describing the influence degree of $C_{i}$ on $C_{j}$.

In this PLFCMs model, we use PLTS to represent the concepts value and the causal relationship between two nodes. Suppose that we have a set of linguistic terms $S = \left\{ {s_{i} } \right\},\;i = - \tau , \ldots ,\tau$ where $\tau$ is a positive number and $s_{i}$ is the possible values of the linguistic terms. Here, we assume that $\tau = 3$, and assign the following meanings to the linguistic terms:$s_{ - 3}$: very strong of negative impact;$s_{ - 2}$: strong of negative impact;$s_{ - 1}$: weak of negative impact;$s_{0}$: undetermined of impact magnitude and direction;$s_{1}$: weak of positive impact;$s_{2}$: strong of positive impact;$s_{3}$: very strong of positive impact. Note the positive/negative impact does not assume positive/negative consequences but the fact the increase or decrease of cause node changes the effect variable in the same/opposite direction.

For example, for the relationship between $C_{1}$ and $C_{2}$, some experts ($e_{1} ,e_{2} ,e_{3}$) are invited to express their opinion by the following statement.$e_{1}$ expresses 60% very strong of negative impact ($s_{ - 3}$) between $C_{1}$ and $C_{2}$, 30% is weak of negative impact ($s_{ - 1}$), and 10% is undetermined about the change magnitude and direction ($s_{0}$). $e_{2}$ expresses 50% very strong of negative impact ($s_{ - 3}$) between $C_{1}$ and $C_{2}$, and 50% is undetermined about the change magnitude and direction ($s_{0}$).$e_{3}$ expresses 40% very strong of negative impact ($s_{ - 3}$) between $C_{1}$ and $C_{2}$, 30% is strong of negative impact ($s_{ - 2}$), and 30% is undetermined about the change magnitude and direction ($s_{0}$). Therefore, the PLE in this case will be $\left\{ {s_{ - 3} (0.5),s_{ - 2} (0.1),s_{ - 1} (0.1),s_{0} (0.3)} \right\}$ when the weights of experts are the same. Similarly, the concept value of nodes can be constructed in the form of a PLE in the same way. Notice that, the number of experts and the number of linguistic terms depends on the actual problem.

In general, to ensure that the node values of each iteration are within the interval [0, 1], a threshold function $f\left( x \right)$ in PLFCMs model with $n$ nodes is required for mapping. And the value of each node at time $t + 1$ can be calculated by the following formula:

$$x_{i} \left( {t + 1} \right) = f\left( {x_{i} \left( t \right) \oplus \sum\limits_{j = 1,j \ne i}^{n} {x_{j} \left( t \right) \otimes \omega_{ji} } } \right)$$

The iteration formula of PLFCMs is

11 $$g\left( {L\left( P \right)} \right)_{i}^{t + 1} = f\left( {g\left( {L\left( P \right)} \right)_{i}^{t} \oplus \sum\limits_{j \in S,j \ne i}^{n} {\left( {g\left( {L\left( P \right)} \right)_{i}^{t} \otimes g\left( {\omega_{ji} \left( p \right)} \right)} \right)} } \right)$$

In this section, we propose a new iteration algorithm by separating membership and its probability as shown in the equations below.

12 $$g(L_{i}^{t + 1} ) = f\left( {g(L_{i}^{t} ) \oplus \sum\limits_{i \ne j,j \in S} {g\left( {L_{i}^{t} \otimes \omega_{ij} } \right)} } \right)$$

13 $$p_{i}^{t + 1} = f\left( {p_{i}^{t} \oplus \sum\limits_{i \ne j,j \in S} {\left( {p_{i}^{t} \otimes p_{ij} } \right)} } \right)$$

In order for an uncertain system to reach a stable state, it is vital to choose a threshold function. When the difference between the two iterations is less than or equal to the given threshold, the iteration can be terminated. In this section, the hyperbolic tangent function [43] is selected as the threshold function $f\left( x \right)$:

14 $$f\left( x \right) = \frac{{e^{\lambda x} - e^{ - \lambda x} }}{{e^{\lambda x} + e^{ - \lambda x} }},\lambda > 0$$

To illustrate that the mapping results of each iteration by $f\left( g \right)$ are still PLEs, we have the following property.

#### Property 2

In PLFCMs $G$, let $\left( {L\left( p \right)} \right)_{i}^{t} = \left( {\left\{ {L_{i}^{1} \left( {p_{1} } \right), \ldots ,L_{i}^{l} \left( {p_{l} } \right)} \right\}} \right)_{i}^{t}$ and $\left( {L\left( p \right)} \right)_{j}^{t} = (\{ L_{j}^{1} \left( {p_{1} } \right), \ldots ,L_{j}^{l}$ $\left( {p_{l} } \right)\} )_{j}^{t}$ are state vectors of concept nodes $C_{1}$ and $C_{2}$ at time $t$ respectively.$\omega_{ij} \left( p \right)$ is the influence degree of concept node $C_{1}$ on $C_{2}$, then the state value $\left( {L\left( p \right)} \right)_{i}^{t + 1}$ at time $t$ is still a PLE. And

$$\begin{aligned} g\left( {L\left( p \right)} \right)_{i}^{{t + 1}} = & g\left( {L\left( p \right)} \right)_{i}^{t} \oplus \sum\limits_{{i \ne j,j \in S}} {\left( {g\left( {L\left( p \right)} \right)_{i}^{t} \otimes g\left( {\omega \left( p \right)} \right)_{{ji}} } \right)} \\ = & \left( {\left\{ {g\left( {L_{i}^{1} \left( {p_{1} } \right)} \right), \ldots ,g\left( {L_{i}^{l} \left( {p_{l} } \right)} \right)} \right\}} \right)_{i}^{t} \\ & \oplus \sum\limits_{{i \ne j,j \in S}} {\left[ {\left( {\left\{ {g\left( {L_{i}^{1} \left( {p_{1} } \right)} \right), \ldots ,g\left( {L_{i}^{l} \left( {p_{l} } \right)} \right)} \right\}} \right)_{i}^{t} } \right.} \\ & \left. { \otimes \left\{ {g\left( {\omega _{{ji}}^{1} (p_{1} )} \right), \ldots ,g\left( {\omega _{{ji}}^{l} (p_{l} )} \right)} \right\}} \right] \\ = & \left\{ {\left[ {g\left( {L_{i}^{k} } \right) + \left( {1 - \prod\limits_{{i \ne j,j \in S}} {\left( {1 - g\left( {L_{i}^{k} } \right) \cdot g\left( {\omega _{{ji}}^{k} } \right)} \right)} } \right)} \right.} \right. \\ & \left. { - g\left( {L_{i}^{k} } \right) \cdot \left( {1 - \prod\limits_{{i \ne j,j \in S}} {\left( {1 - g\left( {L_{i}^{k} } \right) \cdot g\left( {\omega _{{ji}}^{k} } \right)} \right)} } \right)} \right] \\ & \left[ {p_{i}^{k} + \left( {1 - \prod\limits_{{i \ne j,j \in S}} {\left( {1 - p_{i}^{k} \cdot p_{{ji}}^{k} } \right)} } \right)} \right. \\ & \left. {\left. { - p_{i}^{k} \cdot \left( {1 - \prod\limits_{{i \ne j,j \in S}} {\left( {1 - p_{i}^{k} \cdot p_{{ji}}^{k} } \right)} } \right)} \right]} \right\} \\ = & \left\{ {\left[ {1 - \prod\limits_{{i \ne j,j \in S}} {\left( {1 - g\left( {L_{i}^{k} } \right) \cdot g\left( {\omega _{{ji}}^{k} } \right)} \right)} } \right.} \right. \\ & \left. { + g\left( {L_{i}^{k} } \right) \cdot \left( {1 - \prod\limits_{{i \ne j,j \in S}} {\left( {1 - g\left( {L_{i}^{k} } \right) \cdot g\left( {\omega _{{ji}}^{k} } \right)} \right)} } \right)} \right] \\ & \left. {\left[ {1 - \prod\limits_{{i \ne j,j \in S}} {\left( {1 - p_{i}^{k} \cdot p_{{ji}}^{k} } \right)} + p_{i}^{k} \cdot \left( {1 - \prod\limits_{{i \ne j,j \in S}} {\left( {1 - p_{i}^{k} \cdot p_{{ji}}^{k} } \right)} } \right)} \right]} \right\},\,\left( {k = 1,2, \ldots ,n} \right) \\ \end{aligned}$$

Then, we also have $\left( {L\left( P \right)} \right)_{i}^{t + 1} = g^{ - 1} \left( {g\left( {\left( {L\left( P \right)} \right)_{i}^{t + 1} } \right)} \right)$.

If the system reaches a stable state or the iterative result reaches a predetermined threshold, the PLFCMs will stop iterating. In conclusion, the effect of the change in the state value of each node is indicated by probabilistic linguistic term vectors. After several iterations, PLFCMs will reach one of the following states: (1) The state value of node converges to a fixed point, which is called the probabilistic linguistic fixed-point attractor. (2) The state continues to cycle between several fixed states. It is called the probabilistic linguistic limit cycle; (3) It may be confusion, which is useless.

### The analysis method of factors influencing health based on PLFCMs

In this section, we introduce a novel PLFCMs-based method for analyzing factors influencing the health of rural older adults. According to the research of Giabbanelli et al. [47], the expert elicitation process are shown as following. Firstly, determine and invite the corresponding experts. The experts with a high level of research excellence in the field of social security or health economics who are from scientific research institutes and universities will be invited. Then, according to these criteria, a committee of experts is constructed. Their detailed descriptions and characteristics are shown in Table 1. Secondly, obtain indicators systems and cause-effect relationships. One-on-one interviews with experts are used in revising our proposed indicator system; then, questionnaires are sent to experts to obtain the interrelationships between factors. Thirdly, the results obtained from experts are transformed in PLTSs.

**Table 1:** The descriptions and characteristics of experts

Experts	Location	Age	Domain	Title
$E_{1}$	University	30	Social security	Associate professor
$E_{2}$	Research institute	38	Health economic	Professor
$E_{3}$	University	50	Public health	Director
$E_{4}$	University	42	FCMs	Professor

Therefore, the initial state vector with $n + 1$ nodes, which demonstrates the initial value of each concept or node, can be expressed as follows:

$$C^{0} = \{ C_{1} ,C_{2} , \ldots ,C_{n} ,R\}^{0}$$

here $C_{i}^{0} (i = 1,2, \ldots n)$ and $R^{0}$ represent the initial value of concepts and final result, respectively, are denoted by PLTSs.

After identifying the system of factors influencing health, the next step is to identify the interaction between factors and interrelationships between factors and result. Moreover, the causal relationship between two nodes is determined by the knowledge and experience from experts, and the strength of influence is expressed in terms of PLTSs. Therefore, determine the causal connection matrix *W* in which the elements represent the causal relationship or influence strength between concepts.

**Fig. 2:**
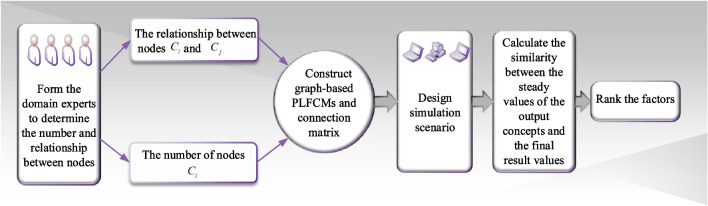
Flowchart of the proposed method

Since the proposed PLFCMs model, the following approach for analyzing the factors affecting the health of rural older adults is put forward, and its flowchart is depicted in Fig. 2. And the specific steps of decision analysis are summarized as below.

- *Step 1* Obtain the nodes of factors affecting the health of rural elderly and the cause-effect relationships between the factors by utilizing the expert elicitation process. In addition, the initial values of each concept are determined by integrating information from the database.
- *Step 2* The relationships between nodes are determined based on the knowledge and experience from experts in accordance with statistical analysis of database and relative literatures about factors influencing health. After obtaining a causal connection matrix, the graph-based PLFCMs model is constructed.
- *Step 3* Design simulation scenario and calculate the initial value of each concept. Using Eqs. (12, 13), we can determine the stable state of concepts.
- *Step 4* According to Eq. (9), the similarity degree of the steady value of concepts and outcomes is calculated, and the primary factors affecting the health of rural older adults are identified.
- *Step 5* End.

## Materials

### Data

The initial state of each variable in this paper is derived from the statistical analysis of variables that are related. We selected samples and variables of rural older adults from the three databases. They are 2014 Chinese Longitudinal Healthy Longevity Survey (CLHLS),^4^ 2015 China Health and Retirement Longitudinal Study (CHARLS),^5^ and 2015 Chinese General Social Survey (CGSS).^6^ The three databases contain demographic, socioeconomic, health, family structure, and lifestyle information, etc., about individuals gleaned from national survey data with random sampling and adequate sample representation (See the Additional file 1 for a detailed introduction).

### Indicator systems

Due to the coupling effect between factors or between factors and health, identifying and intervening in the critical factors affecting health has become the most important and effective strategy for improving the health of rural older adults and achieving healthy aging. Because it is difficult and unrealistic for us to improve health through gene modification and environmental modification in the short term. Therefore, the factors that can be influenced by the Chinese population are primarily medical, lifestyle, and social. China has devoted a substantial number of medical resources to the treatment of diseases over the past few decades. But against the backdrop of rapid aging, the contribution of medical treatment to health improvement is limited. The Chinese government has begun to pay more attention to disease prevention and, in 2019, issued the “Healthy China Initiative”, which advocates for reducing morbidity by promoting healthier lifestyles. Moreover, with the rapid development of China's economy, health disparities resulting from social factors (particularly socioeconomics) have become an issue [48]. Based on the actual situation in China, this paper focuses on *lifestyle*, *social environment*, and *medical factors* as determinants of health. Specific indicator systems are list as follows.

#### Dependent variable: result

Self-rated health is the sole dependent variable in this paper, measuring health status. Self-rated health has been shown to be a valid and reliable indicator for measuring cognitive ability, morbidity, and mortality [3, 3]. It can comprehensively reflect people’s physical and mental health status.

#### Lifestyle and health management variables

We investigate five lifestyle domains: *balanced diet*, *sleeping*, *exercise*, *unhealthy behaviors*, and *mentality*. *A balanced diet* is concerned with eating values and behaviors. *Sleeping* is measured by the quality of one’s sleep. *Exercise* is measured by the presence of physical exercise at present. *Unhealthy behaviors* are reflected by the probability of smoking and drinking. *Mentality* is assessed by the probability of bad emotions lasting for two weeks or more in the previous 12 months.

*Health management* is reflected by the probability of preventive healthcare utilization and the chronic diseases management of rural older adults.

#### Socioeconomic variables

Generally, *income*, *occupation* and *education* are the primary indicators to assess *socioeconomic status*. Instead of a specific household income value, the income is represented by a level to which one’s family economic status belongs in order to more intuitively reflect economic status. In addition, the occupation is measured by the primary occupation of older adults prior to age 60, as people with official job retired at age 60. Education is measured by the self-reported education level.

#### Living environment variables

*Living environment* includes both natural and social environment. Although natural disasters have an impact on people’s health, pollution of natural environmental caused by human behaviors in the process of industrialization has a greater impact on human health than natural disasters. Furthermore, because the social interaction space for rural older adults is limited, *intergenerational relationships* have become an important factor influencing their health. As a result, *air pollution*, *water pollution* and *industrial waste pollution* in the area where the rural older adults living are considered proxy variables of the natural environment, while the frequency of interaction between rural older adults and their children is considered a proxy variable of the social environment.

The specific definition of variables is shown in Table 2.

**Table 2:** The indicator systems of variables

First-level variables	Second-level variables	Indicators	Data sources	References
Health	R Self-rated health	The health status of self-reported	2014 CLHLS	[11]
The lifestyle of the rural older adults	$C_{1}$ Balanced diet	The frequency of eating fresh fruits	2014 CLHLS	[4]
		The frequency of eating fresh vegetables	2014 CLHLS	[4]
	$C_{2}$ Quality of sleep	The sleeping quality of self-reported	2014 CLHLS	[49]
	$C_{3}$ Physical exercise	The presence of physical exercise	2014 CLHLS	[3, 50]
	$C_{4}$ Unhealthy lifestyle	The presence of smoking	2014 CLHLS	[4]
		The presence of drinking	2014 CLHLS	[4]
	$C_{5}$ mentality	The presence of sad, melancholic, or depressed emotion for two weeks or more in last 12 months	2014 CLHLS	[9, 14]
The health management of the rural older adults	$C_{6}$ Preventive medical service utilization	The presence of regular physical examination once every year	2014 CLHLS	[4]
	$C_{7}$ Chronic diseases management	The presence of cooperating with doctors to treat chronic diseases	2015 CHARLS	[51]
Socio-economic status	$C_{8}$ Income	The financial level of the respondents' family	2015 CGSS	[15, 52]
	$C_{9}$ Occupation	The main occupation of respondents before age 60	2014 CLHLS	[4, 53]
	$C_{10}$ Education	The education of respondents	2014 CLHLS	[9, 53]
Living environment	$C_{11}$ Environmental pollution	The severity of air/water/industrial waste pollution in respondents' living areas	2015 CGSS	[54]
	$C_{12}$ Intergenerational relationship	The frequency of respondents to see child	2015 CHARLS	[55]

## Results

### The causal relationship of various factors

**Fig. 3:**
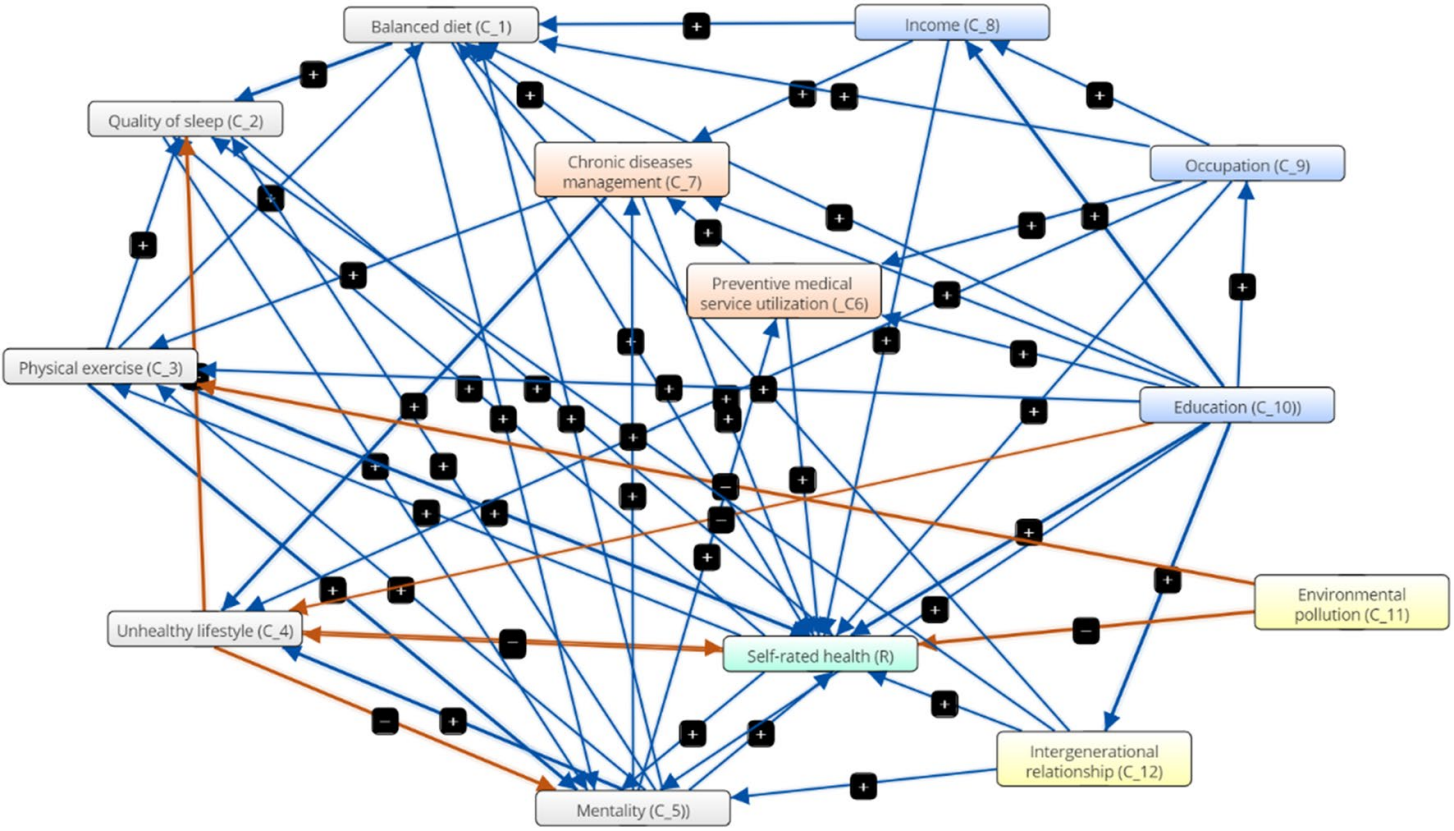
Graph-based causal relationships between factors influencing health of rural older adults

The health status of individual is the result of a combination of various influencing factors. Figure 3 depicts the causal relationships between the various factors as determined by the knowledge and experiences of experts.^7^ The gray box indicates the lifestyle of the rural older adults. The color of orange represents health management of rural older adults. The blue boxes depict socioeconomic status. The yellow box indicates living environment. The green box indicates the health status of rural older adults. Through the causal relationship map, we can find that the factors affecting health are not completely independent. From the perspective of first-level variables, people with higher socioeconomic status are more likely to have healthy lifestyles and a good living environment. At the same time, healthy lifestyles are beneficial to improving health management levels, and a good living environment also aids in the development of a healthy lifestyle. Furthermore, each first-level variable has a relationship with its sub-variables (second-level variables). A balanced diet and a higher sleep quality, for example, must result in improved mental health. Education advancement can help people find better jobs and have a higher income. Otherwise, keep in mind that the causal relationship between various factors can be either positive or negative. Existing research, for example, shows that in China, lifestyle is negatively associated with socioeconomic status, whereas in the United States, lifestyle is positively associated with socioeconomic status [50]. Based on the causal relationships between variables, a connection matrix $W = \left( {\omega_{ij} } \right)_{13 \times 13}$ is obtained, which is shown in Additional file 1: Table S1 (The connection matrix $W$ is the mutual relationship of arbitrary two concepts nodes, and the degree of mutual relationship can be seen intuitively in the matrix.)

### The initial state of each variable

The initial state of all variables, which is obtained by statistical analysis of these variables in the databases, is presented in Table 3. Table 3 demonstrates that approximately 45% of individuals report that they are in good health, while 15% report that they are in poor health. In terms of factors that influence health, more than half of the samples have healthy lifestyles and good health management, while their socioeconomic status and living environment, respectively, are low and ordinary.

**Table 3:** Initial state vector $X_{0}$ with 13 nodes

Second-level variables	Descriptions	Proportion of initial state		
$R$ Self-rated health	The health status of self-reported	Very good (8.78%)	So so (40.38%)	Bad (13.8%)
		Good (35.35%)		Very bad (1.8%)
		$s_{3}$(0.45)	$s_{0}$(0.4)	$s_{ - 3} ($ 0.15)
$C_{1}$ Balanced diet	The frequency of eating fresh fruits and vegetables	Almost every day (12.76%) Quite often (26.06%)	Occasionally (34.84%)	Rarely or never (26.34%)
		Almost every day (55.22%) Quite often (32.64%);	Occasionally (8.06%)	Rarely or never (4.09%)
		$s_{3}$(0.65)	$s_{0}$(0.2)	$s_{ - 3}$(0.15)
$C_{2}$ Quality of sleep	The sleeping quality of self-reported	Very good (15.94%) Good (45.58%);	So so (26.65%)	Bad (10.48%) Very bad (1.35%)
		$s_{3}$(0.6)	$s_{0}$(0.3)	$s_{ - 3}$(0.1)
$C_{3}$ Physical exercise	The presence of physical exercise	Yes (25.45%)	0	No (74.55%)
		$s_{3}$(0.25)	$s_{0}$(0)	$s_{ - 3}$(0.75)
$C_{4}$ Unhealthy lifestyle	The presence of smoking and drinking	Yes (30.48%)	0	No (69.52%)
		Yes (25.56%)	0	No (74.44%)
		$s_{ - 3}$(0.3)	$s_{0}$(0)	$s_{3}$(0.7)
$C_{5}$ Mentality	The presence of sad, melancholic, or depressed emotion for two weeks or more in last 12 months	Yes (12.48%)	0	No (87.52%)
		$s_{ - 3}$(0.15)	$s_{0}$(0)	$s_{3}$(0.85)
$C_{6}$ Preventive medical service utilization	The presence of regular physical examination once every year	Yes (57.7%)	0	No (42.3%)
		$s_{3}$(0.6)	$s_{0}$(0)	$s_{ - 3}$(0.4)
$C_{7}$ Chronic diseases management	The presence of cooperating with doctors to treat chronic diseases	Yes (66.76%)	0	No (33.24%)
		$s_{3}$(0.7)	$s_{0}$(0)	$s_{ - 3}$(0.3)
$C_{8}$ Income	The financial level of the respondents' family	Far below the average (5.86%) Below the average (33.43%)	Average (55.36%)	Above the average (4.69%) Far above the average (0.16%)
		$s_{ - 3}$(0.4)	$s_{0}$(0.55)	$s_{3}$(0.05)
$C_{9}$ Occupation	The main occupation of respondents before age 60	Professional and technical staff (3.18%) Governmental, institutional or manager (2.53%) Commercial, service or industrial worker (8.53%) Self-employed (1.59%) Military personnel (0.74%)	Agriculture, forestry, animal husbandry (75.02%)	Houseworker (6.28%) Never worked (0.54%) Others (1.54%)
		$s_{3}$(0.15)	$s_{0}$(0.75)	$s_{ - 3}$(0.1)
$C_{10}$ Education	The education of respondents	Illiterate (58.15%)	Primary school (30.9%) Junior middle school (6.48%)	Senior middle school (2.59%) College and above (1.88%)
		$s_{ - 3}$(0.6)	$s_{0}$(0.35)	$s_{3}$(0.05)
$C_{11}$ Environmental pollution	The severity of air/water/industrial waste pollution in respondents' living areas	Very serious (9.06%; 9.5%; 6.22%) More serious (16.94%; 20.39%; 14.76%);	Less serious (17.96%; 17.48%; 13.59%) Not serious (22.61%; 20.97%; 18.89%) So so (10.34%; 9.79%; 9.7%) Disinterest (1.75%; 2.02%; 3.71%)	Without pollution (21.31%; 19.81%; 33.08%)
		$s_{ - 3}$(0.3)	$s_{0}$(0.5)	$s_{3}$(0.2)
$C_{12}$ Intergenerational relationship	The frequency of respondents to see child	Almost every day (20.78%) 2–3times a week (5.9%) Once a week (7.07%) Every two weeks (8%) Once a month (10.53%)	Once every three months (10.95%) Once every six months (10.57%) Once a year (19.44%)	Almost never (3.46%) Other (3.31%)
		$s_{3}$(0.5)	$s_{0}$(0.4)	$s_{ - 3}$(0.1)

Generally, the initial state of all variables is presented by 11 granularities that from $s_{ - 5}$ to $s_{5}$. Among them, $s_{ - 5}$ is an extremely poor state,$s_{5}$ is an extremely good state, while $s_{0}$ is the average state or general state. For the convenience of iteration, we select $s_{ - 3}$, $s_{0}$ and $s_{3}$ to present the initial state of variables.

### The steady state after each variable iteration

- *Step 1* Convert linguistic terms information in initial state and adjacency matrix into crisp numbers by Eq. (3) in Def. 4.
- *Step 2* In order to facilitate the simulation, we divide the PLTSs into two parts. The iterative process of all variables are obtained by Eqs.(12) and (13), and shown in Tables 4 and 5, respectively. For convenience, we set the parameter $\lambda = 1$ in Eq. (14), and the threshold value of the iteration result is set to $1 \times 10^{ - 6}$.

**Table 4:** The iteration result of linguistic term in PLTS

Iterations k	k = 1	k = 2	k = 3	$\cdots$	Steady values
[− 3,0,3] → [0.2,0.5,0.8]	[0.6631,0.7582,0.7616]	[0.7610,0.7615,0.7615]	[0.7614,0.7615,0.7615]	$\cdots$	[0.7614,0.7615,0.7615]
[− 3,0,3] → [0.2,0.5,0.8]	[0.6657,0.7582,0.7616]	[0.7610,0.7615,0.7616]	[0.7615,0.7615,0.7616]	$\cdots$	[0.7615,0.7615,0.7616]
[− 3,0,3] → [0.2,0.5,0.8]	[0.6681,0.7593,0.7616]	[0.7611,0.7616,0.7616]	[0.7615,0.7616,0.7616]	$\cdots$	[0.7615,0.7616,0.7616]
[− 3,0,3] → [0.2,0.5,0.8]	[0.6135,0.7506,0.7612]	[0.7584,0.7608,0.7609]	[0.7606,0.7609,0.7609]	$\cdots$	[0.7606,0.7609,0.7609]
[− 3,0,3] → [0.2,0.5,0.8]	[0.6984,0.7609,0.7616]	[0.7615,0.7616,0.7616]	[0.7616,0.7616,0.7616]	$\cdots$	[0.7616,0.7616,0.7616]
[− 3,0,3] → [0.2,0.5,0.8]	[0.6606,0.7573,0.7615]	[0.7609,0.7615,0.7615]	[0.7614,0.7615,0.7615]	$\cdots$	[0.7614,0.7615,0.7615]
[− 3,0,3] → [0.2,0.5,0.8]	[0.6630,0.7581,0.7616]	[0.7609,0.7615,0.7616]	[0.7614,0.7615,0.7616]	$\cdots$	[0.7614,0.7615,0.7616]
[− 3,0,3] → [0.2,0.5,0.8]	[0.6495,0.7562,0.7615]	[0.7605,0.7614,0.7615]	[0.7613,0.7614,0.7615]	$\cdots$	[0.7613,0.7614,0.7615]
[− 3,0,3] → [0.2,0.5,0.8]	[0.6775,0.7598,0.7616]	[0.7613,0.7616,0.7616]	[0.7615,0.7616,0.7616]	$\cdots$	[0.7615,0.7616,0.7616]
[− 3,0,3] → [0.2,0.5,0.8]	[0.6877,0.7608,0.7616]	[0.7614,0.7616,0.7616]	[0.7616,0.7616,0.7616]	$\cdots$	[0.7616,0.7616,0.7616]
[− 3,0,3] → [0.2,0.5,0.8]	[0.6404,0.7518,0.7611]	[0.7600,0.7610,0.7608]	[0.7614,0.7615,0.7615]	$\cdots$	[0.7611,0.7610,0.7608]
[− 3,0,3] → [0.2,0.5,0.8]	[0.6731,0.7597,0.7616]	[0.7612,0.7616,0.7616]	[0.7615,0.7616,0.7616]	$\cdots$	[0.7615,0.7616,0.7616]
[− 3,0,3] → [0.2,0.5,0.8]	[0.6607,0.7584,0.7616]	[0.7609,0.7615,0.7616]	[0.7614,0.7615,0.7616]	$\cdots$	[0.7614,0.7615,0.7616]

**Table 5:** the iteration result of probability associated with linguistic term in PLTS

Initial values	k = 1	k = 2	k = 3	$\cdots$	Steady values
[0.15,0.2,0.65]	[0.2190,0.3417,0.4393]	[0.3340,0.3530,0.3130]	[0.3891,0.3719,0.2390]	$\cdots$	[0.4826,0.4708,0.0466]
[0.1,0.3,0.6]	[0.2895,0.2760,0.4344]	[0.3891,0.2832,0.3277]	[0.4201,0.3126,0.2673]	$\cdots$	[0.4972,0.4199,0.0829]
[0.75,0,0.25]	[0.4860,0.2100,0.3040]	[0.4078,0.3128,0.2794]	[0.4008,0.3518,0.2474]	$\cdots$	[0.4659,0.4415,0.0926]
[0.3,0,0.7]	[0.2872,0.2467,0.4661]	[0.3341,0.3376,0.3283]	[0.3676,0.3852,0.2472]	$\cdots$	[0.4620,0.4905,0.0474]
[0.15,0,0.85]	[0.3176,0.2620,0.4204]	[0.3411,0.3624,0.2965]	[0.3581,0.3988,0.2431]	$\cdots$	[0.4446,0.4850,0.0704]
[0.4,0,0.6]	[0.3195,0.2331,0.4474]	[0.2984,0.3349,0.3666]	[0.2887,0.4037,0.3076]	$\cdots$	[0.3805,0.5518,0.0677]
[0.3,0,0.7]	[0.3849,0.1871,0.4280]	[0.3870,0.3114,0.3015]	[0.3988,0.3703,0.2309]	$\cdots$	[0.4794,0.4744,0.0462]
[0.4,0.55,0.05]	[0.4269,0.4806,0.0925]	[0.4511,0.4458,0.1031]	[0.4607,0.4397,0.0997]	$\cdots$	[0.4998,0.4852,0.0150]
[0.1,0.75,0.15]	[0.3019,0.4969,0.2012]	[0.3568,0.4348,0.2084]	[0.3738,0.4261,0.2001]	$\cdots$	[0.4428,0.4865,0.0707]
[0.6,0.35,0.05]	[0.3716,0.3650,0.2634]	[0.3625,0.3839,0.2536]	[0.3714,0.3963,0.2323]	$\cdots$	[0.4402,0.4586,0.1012]
[0.3,0.5,0.2]	[0.3641,0.3698,0.2661]	[0.3609,0.3360,0.3031]	[0.3492,0.3412,0.3096]	$\cdots$	[0.3942,0.4294,0.1764]
[0.1,0.4,0.5]	[0.1657,0.3832,0.4511]	[0.2454,0.3722,0.3823]	[0.2915,0.3729,0.3356]	$\cdots$	[0.3895,0.4449,0.1656]
[0.45,0.4,0.15]	[0.2973,0.2905,0.4122]	[0.3254,0.3228,0.3518]	[0.3356,0.3599,0.3045]	$\cdots$	[0.4240,0.4643,0.1118]

### The rank of factors influencing the health of rural older adults

Firstly, before calculating the similarity between concepts nodes, we concert the iteration result into PLTSs by Eq. (4). Secondly, the distance of two PLTSs can be obtained by Eq. (8). Then, the similarity degrees between the steady values of factors and the health of rural older adults are calculated by Eq. (9), where the Z mapping function is $Z\left( t \right) = \frac{1 - t}{{1 + t}}$. The results are shown as below.

$$\begin{aligned} & S\left( {C_{1} ,R} \right) = 0.8577;\quad S\left( {C_{2} ,R} \right) = 0.8538; \\ & S\left( {C_{3} ,R} \right) = 0.9141;\quad S\left( {C_{4} ,R} \right) = 0.8723; \\ & S\left( {C_{5} ,R} \right) = 0.9151;\quad S\left( {C_{6} ,R} \right) = 0.8293; \\ & S\left( {C_{7} ,R} \right) = 0.8598;\quad S\left( {C_{8} ,R} \right) = 0.8043; \\ & S\left( {C_{9} ,R} \right) = 0.9157;\quad S\left( {C_{{10}} ,R} \right) = 0.9652; \\ & S\left( {C_{{11}} ,R} \right) = 0.8706;\quad S\left( {C_{{12}} ,R} \right) = 0.8901. \\ \end{aligned}$$

As shown in Table 6, various health-influencing factors have distinct effects on the health of rural older adults. Overall, *socioeconomic status*, *the living environment*, *lifestyle*, and *health management* have the greatest, second-greatest, third-greatest, and fourth-greatest impact on health, in that order. *Education*, *occupation*, *mentality*, *physical exercise* and *intergenerational relationship* are the top five second-level indicators that have the greatest impact on the health of rural older adults. In general, *lifestyle* and *environment* are the most important factors influencing health. However, each of them contains several secondary factors, and these secondary factors may have varying effects on different groups. Only the two lifestyles of *physical exercise* and *mentality* have greater effects on the health of rural older adults, according to our findings. The effects of *a balanced diet* and *sleeping quality* on the health of rural older adults are minimal. Contrary to common sense, *education* and *occupation* in the *socioeconomic status* have the greatest effects on the health of rural older adults, while the *family economic status* has the least effect. In addition, the effects of preventive healthcare utilization and chronic disease management are minimal.

**Table 6:** the similarity ranking of health influencing factors

First-level indicators	Second-level indicators	Ranking	Total score of ranking
The lifestyle of the rural older adults	$C_{1}$ Balanced diet	9	(9 + 10 + 4 + 6 + 3)/5 = 6.4
	$C_{2}$ The quality of sleep	10	
	$C_{3}$ Physical exercise	4	
	$C_{4}$ Unhealthy lifestyle	6	
	$C_{5}$ Mentality	3	
The health management	$C_{6}$ Preventive medical service utilization	11	(8 + 11)/2 = 9.5
	$C_{7}$ Chronic disease management	8	
Socio-economic status	$C_{8}$ Income	12	(12 + 2 + 1)/3 = 5
	$C_{9}$ Occupation	2	
	$C_{10}$ Education	1	
Living environment	$C_{11}$ Environmental pollution	7	(7 + 5)/2 = 6
	$C_{12}$ Intergenerational relationship	5	

To achieve healthy ageing, precise intervention in the critical factors affecting health of rural older adults is required. Using the PLFCMs, this paper analyzes the factors affecting the health of rural older adults and identifies the most critical factors affecting the health of rural older adults in China. In accordance with the present results, previous studies have demonstrated that *education* has a very significant positive impact on the health of older adults [7, 7, 7]. Moreover, with the increase in *age*, *mentality*, *physical exercise*, and *family intergenerational relationships* have gradually become the critical factors that affect the health of rural older adults [13, 13]. Other health-influencing factors, such as *smoking*, *drinking*, *environmental pollution*, *chronic disease management*, etc., have a negligible impact on the health of rural older adults. The reasons may be that the effect degree of some health- influencing factors varies regions and age groups. On the one hand, as rural older adults’ physical function declines and health capital depreciation accelerates, they may begin to doubt their social value, causing psychological problems. Meanwhile, physical exercise also becomes a critical factor to increase their health stock. Rural older adults, on the other hand, become increasingly reliant on their children as they age. However, a large number of young and middle-aged people in rural areas choose to go out to work (migrant workers), increasing the number of older adults who are left behind. As a result, the impact of *intergenerational relationships* on health is growing among rural older adults.

Another interesting finding is that the *primary occupation* of rural older adults prior to the age of 60 has a great effect on their current health status. Most previous studies on older adults ignored the relationship between their occupation and health because assumed that older adults were no longer working, and they placed more emphasis on occupation-related pension and pension insurance. However, this is not the case for rural older adults. According to the data analysis in this paper, only about 16% of the rural older adults have a regular occupation before the age of 60, and approximately 75% of them work in agriculture, forestry, and other fields. As a result, most rural older adults do not receive employer-provided pension benefits, and their previous manual labor experiences are also closely related to their current health status.

What is surprising is that *family income* status has the least impact on the health of rural older adults, which appears to contradict the previous research findings. Generally, *family income* is an important material basis for maintaining and improving the health of the older adults as a measure of *socioeconomic status* [9, 9, 9]. Due to their strong payment capacity, high-income groups can purchase more nutritional foods and receive superior healthcare services, for instance. However, the finding of this paper shows that *family income* is the least influential factor on the health of rural older adults. This may be because *family income* mainly affects the health of rural older adults by altering their *lifestyle* and *living environment*, and its indirect impact on health is significantly greater than its direct impact. Another possible explanation for this might be that the income of rural older adults is mainly derived from their labor and children’s support, and lack the authority to make decisions regarding family income. Consequently, despite the fact that there is a correlation between family income and the health of rural older adults, the relationship between the two is very weak.

### Sensitivity analysis

In this part, sensitivity analyses on the different forms of $Z\left( t \right)$ and different $\lambda$ are conducted, respectively.

#### The different forms of $Z\left( t \right)$

In the similarity measure of PLTSs, there are several usual forms of $Z\left( t \right)$ in Def. 6, i.e. (1) $Z\left( t \right) = 1 - t$; (2) $Z(t) = \frac{1 - t}{{1 + t}}$; (3) $Z(t) = 1 - te^{t - 1}$; (4) $Z(t) = 1 - t^{2}$. To illustrate the robust of results, a comparison analysis for them is provided in this part and is shown in Table 7.

**Table 7:** the comparisons of different forms of $Z\left( t \right)$

Factors	$Z(t) = 1 - t$		$Z(t) = \frac{1 - t}{{1 + t}}$		$Z(t) = 1 - te^{t - 1}$		$Z(t) = 1 - t^{2}$	
	Similarity	Ranks	Similarity	Ranks	Similarity	Ranks	Similarity	Ranks
$C_{1}$	0.9234	9	0.8577	9	0.9696	9	0.9941	9
$C_{2}$	0.9212	10	0.8538	10	0.9686	10	0.9938	10
$C_{3}$	0.9551	4	0.9141	4	0.9827	4	0.9980	4
$C_{4}$	0.9318	6	0.8723	6	0.9731	6	0.9953	6
$C_{5}$	0.9557	3	0.9151	3	0.9829	3	0.9980	3
$C_{6}$	0.9067	11	0.8293	11	0.9623	11	0.9913	11
$C_{7}$	0.9246	8	0.8598	8	0.9701	8	0.9943	8
$C_{8}$	0.8915	12	0.8043	12	0.9555	12	0.9882	12
$C_{9}$	0.9560	2	0.9157	2	0.9831	2	0.9981	2
$C_{10}$	0.9823	1	0.9652	1	0.9934	1	0.9997	1
$C_{11}$	0.9308	7	0.8705	7	0.9727	7	0.9952	7
$C_{12}$	0.9419	5	0.8901	5	0.9773	5	0.9966	5

From the above calculations, we find that the ranks obtained by different forms of $Z\left( t \right)$ are the same, that is, the most important factor affecting the health of rural older adults is $C_{10}$. Thus, the result is robust, that is, it is not disturbed by the mapping function forms of $Z\left( t \right)$.

#### The sensitivity analysis of $\lambda$

The threshold function in the iteration process of the cause–effect analysis is a hyperbolic tangent function. Table 8 shows that results obtained by different parameter $\lambda$.

**Table 8:** different ranking results with different $\lambda$

$\lambda = 2$		$\lambda = 3$		$\lambda = 4$		$\lambda = 6$		$\lambda = 8$		$\lambda = 10$	
Similarity	Factors	Similarity	Factors	Similarity	Factors	Similarity	Factors	Similarity	Factors	Similarity	Factors
0.6346	$C_{8}$	0.8071	$C_{8}$	0.9049	$C_{8}$	0.9766	$C_{8}$	0.9942	$C_{8}$	0.9986	$C_{8}$
0.7467	$C_{1}$	0.8590	$C_{1}$	0.9299	$C_{1}$	0.9833	$C_{1}$	0.9961	$C_{1}$	0.9991	$C_{1}$
0.7539	$C_{4}$	0.8625	$C_{4}$	0.9313	$C_{4}$	0.9835	$C_{4}$	0.9961	$C_{4}$	0.9991	$C_{4}$
0.7642	$C_{7}$	0.8775	$C_{7}$	0.9424	$C_{7}$	0.9876	$C_{7}$	0.9974	$C_{7}$	0.9994	$C_{7}$
0.8393	$C_{6}$	0.9145	$C_{6}$	0.9571	$C_{6}$	0.9898	$C_{6}$	0.9977	$C_{6}$	0.9995	$C_{6}$
0.8397	$C_{2}$	0.9154	$C_{2}$	0.9604	$C_{2}$	0.9918	$C_{2}$	0.9983	$C_{2}$	0.9997	$C_{2}$
0.8785	$C_{3}$	0.9339	$C_{3}$	0.9694	$C_{3}$	0.9941	$C_{3}$	0.9989	$C_{3}$	0.9998	$C_{3}$
0.8810	$C_{5}$	0.9471	$C_{5}$	0.9789	$C_{5}$	0.9968	$C_{5}$	0.9995	$C_{5}$	0.9999	$C_{9}$
0.8943	$C_{9}$	0.9555	$C_{9}$	0.9817	$C_{9}$	0.9970	$C_{9}$	0.9995	$C_{9}$	0.9999	$C_{5}$
0.9082	$C_{11}$	0.9679	$C_{11}$	0.9884	$C_{11}$	0.9982	$C_{12}$	0.9997	$C_{11}$	0.9999	$C_{11}$
0.9193	$C_{12}$	0.9696	$C_{12}$	0.9887	$C_{12}$	0.9983	$C_{11}$	0.9997	$C_{12}$	1.0000	$C_{12}$
0.9562	$C_{10}$	0.9800	$C_{10}$	0.9926	$C_{10}$	0.9991	$C_{10}$	0.9999	$C_{10}$	1.0000	$C_{10}$

**Fig. 4:**
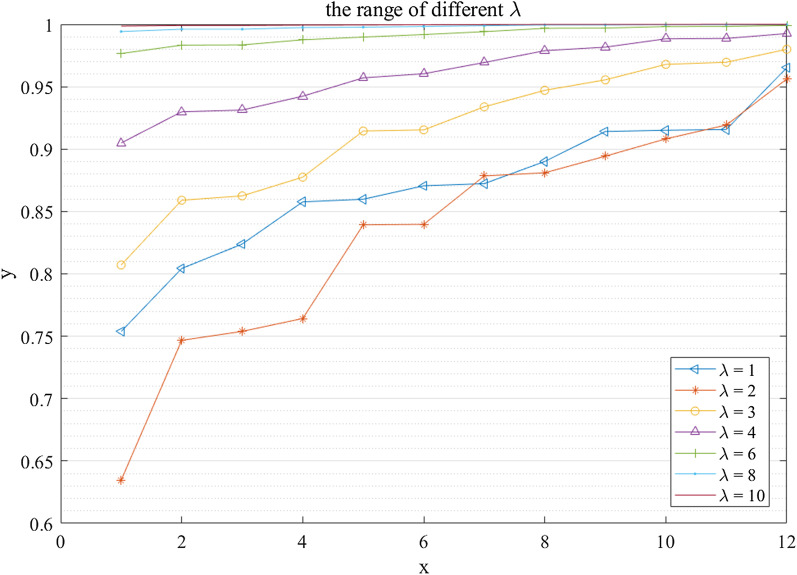
The intuitive representation of the change with different $\lambda$

From the above results shown in Table 8, we can find that the most important factor is the same *i.e.*, $C_{10}$(*Education*). The worst factors with different $\lambda$ all are $C_{8}$ (*Income*). When $\lambda = 6$, the position of $C_{11}$ and $C_{12}$ are different with that obtained by other parameters $\lambda$. And the ranking results of $C_{5}$ and $C_{9}$ are different with other results when $\lambda = 10$. Besides, the importance of $C_{11}$ and $C_{12}$ is the same. As parameter $\lambda$ varies from 2 to 10, the range of similarity degrees becomes smaller. The intuitive representation of the change of range is shown in Fig. 4. From the results, we can find that the highest distinction of factors is reached when $\lambda = 2$. Although $\lambda = 1$ is selected in the cause–effect analysis of the health of rural older adults, the ranking results are also the same with that obtained with $\lambda = 2$. It does not affect the final results of the factor analysis.

### Comparison analyses

#### Compare with the HFLCMs

As an extension of FCMs, the hesitant fuzzy linguistic cognitive maps (HFLCMs) model has been applied in solar energy generation [32]. However, because it disregards the probabilities of linguistic terms, it fails to represent the varying probability of linguistic evaluation obtained from experts or historical data. In contrast, our proposed model can effectively overcome this problem. Not only can it provide comprehensive evaluations, but it can also portray their probabilities of them. And when the probability of linguistic term equals to 1, the PLTSs could degenerate into HFLTSs. Therefore, the PLFCMs degrades to HFLCMs model if the probability information of linguistic evaluation equals to 1. And the computational procedures and outcomes are shown as follows:

- *Step 1* To facilitate comparison, the probabilities associated with linguistic terms are disregarded when the PLEs are transformed in a hesitant fuzzy linguistic elements. And the results are shown in Additional file 1: Table S2.
- *Step 2* The iterative results obtained by HFLCMs are identical to the results shown in Table 4.
- *Step 3* The similarity measures between the steady values of factors affecting the health of rural older adults and health outcomes are calculated Eq. (9), as follow Table 9.
- *Step 4* End.

**Table 9:** The similarity results of HFLCMs model

Factors	Similarity
$C_{4}$	0.9919
$C_{11}$	0.9937
$C_{8}$	0.9985
$C_{5}$	0.9988
$C_{10}$	0.9989
$C_{9}$	0.9992
$C_{12}$	0.9993
$C_{6}$	0.9995
$C_{3}$	0.9996
$C_{2}$	0.9997
$C_{1}$	0.9998
$C_{7}$	0.9999

According to the findings of HFLCMs, the most important factors affecting the health of rural older adults are *Chronic diseases management*, *Balanced diet*, *Quality of sleep*, and so on. We can see that the results differ from those obtained by the proposed method. This distinction is made for a variety of reasons. To begin, the similarity degrees range from 0.9919 to 0.9999, and the values are insufficient to distinguish the difference between factors. Whereas the similarity degree obtained by our proposed can distinguish the difference between factors well. Secondly, our proposed novel PLFCMs model can consider the probability of linguistic evaluations. According to the above comparison of PLFCMs and other forms of FCMs shown in a literature review, PLFCMs has more advantages in expressing uncertainty. It can effectively deal with the uncertainty of experts and accurately reflect different group opinions. Thus, PLFCMs outperforms the HFLCMs when modeling complex systems. And the PLFCMs model produces more reasonable and reliable results.

#### Compare with the conventional FCMs

To illustrate the superiority of the proposed model, a comparison with FCMs model is shown in this section. The PLFCMs degrades to FCMs model where the adjacency matrix is replaced by score degree of PLTS. And the computational steps and results are shown as follows:

- *Step 1* To facilitate comparison, the PLEs are transformed in a score degree. And the results are shown in Additional file 1: Table S3.
- *Step 2* The iterative final results obtained by FCMs are shown in Table 10.
- *Step 3* According to Eq. (9), the similarity measures between the steady values of factors affecting the health of rural older adults and health outcome are calculated as follow Table 11.

**Table 10:** The iteration result of FCMs

Iterations k	k = 1	k = 2	k = 3	$\cdots$	Steady values
0.6500	0.96887	0.99587	0.99653	$\cdots$	0.99654
0.6500	0.97365	0.99689	0.99733	$\cdots$	0.99734
0.3500	0.88611	0.99836	0.99933	$\cdots$	0.99934
0.6200	0.74747	0.82300	0.85752	$\cdots$	0.87896
0.7100	0.99990	1.00000	1.00000	$\cdots$	1.00000
0.5600	0.89577	0.98087	0.98765	$\cdots$	0.98810
0.6200	0.97778	0.99832	0.99855	$\cdots$	0.99855
0.3950	0.77429	0.96547	0.98713	$\cdots$	0.98859
0.5150	0.98799	0.99989	0.99990	$\cdots$	0.99990
0.3350	0.99142	1.00000	1.00000	$\cdots$	1.00000
0.4700	0.57703	0.66840	0.73327	$\cdots$	0.81453
0.6200	0.99311	0.99977	0.99978	$\cdots$	0.99979
0.5900	0.97661	0.99871	0.99891	$\cdots$	0.99891

**Table 11:** The similarity results of FCMs model

Factors	Similarity
$C_{11}$	0.6886
$C_{4}$	0.7858
$C_{6}$	0.9786
$C_{8}$	0.9796
$C_{1}$	0.9953
$C_{2}$	0.9969
$C_{10}$	0.9978
$C_{5}$	0.9978
$C_{9}$	0.9980
$C_{12}$	0.9982
$C_{3}$	0.9991
$C_{7}$	0.9993

We can find that the results obtained by FCMs model is different with our proposed method. The most important factors affecting the health of rural older adults are *Chronic diseases management*, *Physical exercise*, *Intergenerational relationship*, and so on. There are some reasons for this variation. Firstly, the data of FCMs model is degraded from PLTSs, and some important information may be lost in the transformation process. Secondly, the similarity degrees of some factors vary from 0.9953 to 0.9993, and the values are insufficient to distinguish the difference among factors. Thus, PLFCMs is better than FCMs in modeling the uncertainty of complex system. And the results obtained by the proposed model is more reasonable and reliable than FCMs.

## Conclusion and policy implication

Only by achieving healthy aging can China and the rest of the world alleviate the pressures of aging. In this paper, a novel extension of FCMs model called PLFCMs is proposed, and the complete mathematical model is established. In addition, some sensitivity and comparations analysis are illustrated to show the superiority of proposed method. And a real-world case for analyzing the health influencing factors of rural older adults is solved by the proposed method. The results show that *education* and *occupation* are the most critical factors affecting the health of rural older adults, followed by *mentality*, *physical exercise*, and *intergenerational relationships*. These results suggest that the effect degree of each health factor will change across different regions and different age groups. Therefore, distinct health interventions should be implemented for different groups. For the rural older adults, it is currently difficult to maintain or improve their health by improving education and occupational status. Nonetheless, the government can provide more spiritual solace to the rural older adults by cultivating social organizations and purchasing social services. In the meantime, it can also encourage the rural older adults to engage in appropriate physical exercises by constructing village sports venues and facilities and by promoting a “filial piety” culture that fosters harmonious intergenerational family relationship. What’s more, enhancing the education of social members and bolstering occupational health management remain critical preventive measures for the health of rural older adults. The codes of this article are shown in Additional File 2.

## Supplementary Information

**Additional file 1**. Supplementary.

**Additional file 2**. The codes of this article.

## Data Availability

All data generated or analyzed during this study are included in this published article.

## Footnotes

1 The data is from the National Bureau of Statistics of China.

2 The data is from Analysis Report of National Health Services Survey in China, 2013.

3 The data is from Chinese National Health and Nutrition Big Data. http://www.sohu.com/a/252677908_781627.

4 https://opendata.pku.edu.cn/dataset.xhtml?persistentId=doi:10.18170/DVN/XRV2WN.

5 http://charls.pku.edu.cn/pages/data/111/zh-cn.html.

6 http://cnsda.ruc.edu.cn/index.php?r=projects/view&id=62072446.

7 The figure is drawn by the authors with the help of the website https://www.mentalmodeler.com/scenario/.
