# Supplementary material for: Probabilistic linguistic fuzzy cognitive maps: applications to the critical factors affecting the health of rural older adults
Source: BMC Med Inform Decis Mak. 2022 Nov 17;22:299. doi: 10.1186/s12911-022-02028-9 (PMC9673458; doi:10.1186/s12911-022-02028-9)
Supplement: Supplementary file 1 — Additional file 1. Supplementary. [file 12911_2022_2028_MOESM1_ESM.docx]

**Supplementary Material**

**The introduction of database**

Chinese Longitudinal Healthy Longevity Survey (CLHLS) is conducted by the Health Ageing and Development Research Center of Peking University. It covers 23 provinces/autonomous regions/municipalities across the country and its respondents are the elderly who are aged 60 and above. China Health and Retirement Longitudinal Study (CHARLS) is also a high-quality database about individuals who are aged 45 and above, and the database were established by the National Development Research Institute of Peking University. It covers around 12,400 households and 2,3000 interviewees in different regions of China by using the method of population proportion sampling (PPS), and 450 communities of 150 counties in 28 provinces were interviewed in this investigation. Chinese General Social Survey (CGSS) is the earliest national, comprehensive, and continuous academic survey project in China. It is executed by the China Survey and Data Center of Renmin University of China and comprehensively collects data on multiple levels of society, community, family and individual.

The data to this paper can be downloaded with reasonable applications online at <https://opendata.pku.edu.cn/dataset.xhtml?persistentId=doi:10.18170/DVN/XRV2WN>(CLHLS 2014);

<http://charls.pku.edu.cn/pages/data/111/zh-cn.html>(CHARLS2015); <http://cnsda.ruc.edu.cn/index.php?r=projects/view&id=62072446> (CGSS 2015)

**Table S1** Strength of association between cause factors of PLFCM model

| $\omega_{ij}$ | $C_{1}$ | $C_{2}$ | $C_{3}$ | $C_{4}$ | $C_{5}$ | $C_{6}$ | $C_{7}$ | $C_{8}$ | $C_{9}$ | $C_{10}$ | $C_{11}$ | $C_{12}$ | $R$ |
| --- | --- | --- | --- | --- | --- | --- | --- | --- | --- | --- | --- | --- | --- |
| $C_{1}$ | {$s_{0}$(0)} | {$s_{1}$(0.5),$s_{2}$(0.3),$s_{3}$(0.2)} | {$s_{0}$(0)} | {$s_{0}$(0)} | {$s_{1}$(0.7),$s_{2}$(0.2)} | {$s_{0}$(0)} | {$s_{0}$(0)} | {$s_{0}$(0)} | {$s_{0}$(0)} | {$s_{0}$(0)} | {$s_{0}$(0)} | {$s_{0}$(0)} | {$s_{4}$(0.2),$s_{5}$(0.8)} |
| $C_{2}$ | {$s_{0}$(0)} | {$s_{0}$(0)} | {$s_{0}$(0)} | {$s_{0}$(0)} | {$s_{3}$(0.2),$s_{4}$(0.5),$s_{5}$(0.3)} | {$s_{0}$(0)} | {$s_{1}$(0.8)} | {$s_{0}$(0)} | {$s_{0}$(0)} | {$s_{0}$(0)} | {$s_{0}$(0)} | {$s_{0}$(0)} | {$s_{3}$(0.5),$s_{4}$(0.4),$s_{5}$(0.1)} |
| $C_{3}$ | {$s_{1}$(0.5),$s_{2}$(0.4),$s_{3}$(0.1)} | {$s_{2}$(0.3),$s_{3}$(0.4),$s_{4}$(0.2)} | {$s_{0}$(0)} | {$s_{0}$(0)} | {$s_{4}$(0.6),$s_{5}$(0.4)} | {$s_{0}$(0)} | {$s_{0}$(0)} | {$s_{0}$(0)} | {$s_{0}$(0)} | {$s_{0}$(0)} | {$s_{0}$(0)} | {$s_{0}$(0)} | {$s_{3}$(0.5),$s_{4}$(0.3),$s_{5}$(0.2)} |
| $C_{4}$ | {$s_{0}$(0)} | {$s_{-4}$(0.5),$s_{-3}$(0.3),$s_{-2}$(0.2)} | {$s_{0}$(0)} | {$s_{0}$(0)} | {$s_{-2}$(0.4),$s_{-1}$(0.5)} | {$s_{0}$(0)} | {$s_{0}$(0)} | {$s_{0}$(0)} | {$s_{0}$(0)} | {$s_{0}$(0)} | {$s_{0}$(0)} | {$s_{0}$(0)} | {$s_{-5}$(0.3),$s_{-4}$(0.7)} |
| $C_{5}$ | {$s_{3}$(0.2),$s_{4}$(0.6),$s_{5}$(0.1)} | {$s_{4}$(0.2),$s_{5}$(0.7)} | {$s_{1}$(0.3),$s_{2}$(0.6),$s_{3}$(0.1)} | {$s_{3}$(0.2),$s_{4}$(0.4),$s_{5}$(0.4)} | {$s_{0}$(0)} | {$s_{3}$(0.4),$s_{4}$(0.5),$s_{5}$(0.1)} | {$s_{4}$(0.5),$s_{5}$(0.5)} | {$s_{0}$(0)} | {$s_{0}$(0)} | {$s_{0}$(0)} | {$s_{0}$(0)} | {$s_{0}$(0)} | {$s_{4}$(0.4),$s_{5}$(0.5)} |
| $C_{6}$ | {$s_{0}$(0)} | {$s_{0}$(0)} | {$s_{0}$(0)} | {$s_{0}$(0)} | {$s_{0}$(0)} | {$s_{0}$(0)} | {$s_{3}$(0.2),$s_{4}$(0.5),$s_{5}$(0.3)} | {$s_{0}$(0)} | {$s_{0}$(0)} | {$s_{0}$(0)} | {$s_{0}$(0)} | {$s_{0}$(0)} | {$s_{1}$(0.2),$s_{2}$(0.8)} |
| $C_{7}$ | {$s_{1}$(0.5),$s_{2}$(0.4),$s_{3}$(0.1)} | {$s_{0}$(0)} | {$s_{1}$(0.4),$s_{2}$(0.5),$s_{4}$(0.1)} | {$s_{2}$(0.5),$s_{3}$(0.3),$s_{5}$(0.1)} | {$s_{0}$(0)} | {$s_{0}$(0)} | {$s_{0}$(0)} | {$s_{0}$(0)} | {$s_{0}$(0)} | {$s_{0}$(0)} | {$s_{0}$(0)} | {$s_{0}$(0)} | {$s_{1}$(0.4),$s_{2}$(0.5)} |
| $C_{8}$ | {$s_{-2}$(0.5),$s_{-1}$(0.4),$s_{2}$(0.1)} | {$s_{0}$(0)} | {$s_{0}$(0)} | {$s_{0}$(0)} | {$s_{0}$(0)} | {$s_{0}$(0)} | {$s_{1}$(0.5),$s_{2}$(0.5)} | {$s_{0}$(0)} | {$s_{0}$(0)} | {$s_{0}$(0)} | {$s_{0}$(0)} | {$s_{0}$(0)} | {$s_{1}$(0.5),$s_{2}$(0.4)} |
| $C_{9}$ | {$s_{1}$(0.3),$s_{2}$(0.5)} | {$s_{0}$(0)} | {$s_{0}$(0)} | {$s_{4}$(0.3),$s_{5}$(0.7)} | {$s_{0}$(0)} | {$s_{1}$(0.4),$s_{2}$(0.5),$s_{5}$(0.1)} | {$s_{0}$(0)} | {$s_{3}$(0.2),$s_{4}$(0.4),$s_{5}$(0.4)} | {$s_{0}$(0)} | {$s_{0}$(0)} | {$s_{0}$(0)} | {$s_{0}$(0)} | {$s_{3}$(0.5),$s_{4}$(0.3),$s_{5}$(0.2)} |
| $C_{10}$ | {$s_{2}$(0.3),$s_{3}$(0.5),$s_{5}$(0.2)} | {$s_{0}$(0)} | {$s_{1}$(0.6),$s_{2}$(0.3),$s_{4}$(0.1)} | {$s_{1}$(0.3),$s_{2}$(0.5)} | {$s_{0}$(0.2),$s_{2}$(0.3),$s_{4}$(0.5)} | {$s_{1}$(0.4),$s_{3}$(0.5)} | {$s_{2}$(0.3),$s_{3}$(0.5)} | {$s_{3}$(0.2),$s_{4}$(0.7)} | {$s_{1}$(0.2),$s_{4}$(0.5),$s_{5}$(0.3)} | {$s_{0}$(0)} | {$s_{0}$(0)} | {$s_{4}$(0.5),$s_{5}$(0.5)} | {$s_{3}$(0.4),$s_{4}$(0.5)} |
| $C_{11}$ | {$s_{0}$(0)} | {$s_{0}$(0)} | {$s_{2}$(0.5),$s_{3}$(0.4),$s_{5}$(0.1)} | {$s_{0}$(0)} | {$s_{0}$(0)} | {$s_{0}$(0)} | {$s_{0}$(0)} | {$s_{0}$(0)} | {$s_{0}$(0)} | {$s_{0}$(0)} | {$s_{0}$(0)} | {$s_{0}$(0)} | {$s_{1}$(0.1),$s_{3}$(0.4),$s_{4}$(0.5)} |
| $C_{12}$ | {$s_{3}$(0.4),$s_{4}$(0.5),$s_{5}$(0.1)} | {$s_{1}$(0.1),$s_{3}$(0.4),$s_{4}$(0.5)} | {$s_{0}$(0)} | {$s_{0}$(0)} | {$s_{4}$(0.5),$s_{5}$(0.5)} | {$s_{0}$(0)} | {$s_{0}$(0)} | {$s_{0}$(0)} | {$s_{0}$(0)} | {$s_{0}$(0)} | {$s_{0}$(0)} | {$s_{0}$(0)} | {$s_{1}$(0.1),$s_{4}$(0.3),$s_{5}$(0.5)} |
| $R$ | {$s_{0}$(0)} | {$s_{2}$(0.3),$s_{3}$(0.4),$s_{4}$(0.3)} | {$s_{2}$(0.3),$s_{3}$(0.5)} | {$s_{-2}$(0.3),$s_{1}$(0.2),$s_{2}$(0.5)} | {$s_{2}$(0.3),$s_{3}$(0.6),$s_{5}$(0.1)} | {$s_{0}$(0)} | {$s_{0}$(0)} | {$s_{0}$(0)} | {$s_{0}$(0)} | {$s_{0}$(0)} | {$s_{0}$(0)} | {$s_{0}$(0)} | {$s_{0}$(0)} |

**Table S2** Strength of association between cause factors of HFLCM model

| $\omega_{ij}$ | $C_{1}$ | $C_{2}$ | $C_{3}$ | $C_{4}$ | $C_{5}$ | $C_{6}$ | $C_{7}$ | $C_{8}$ | $C_{9}$ | $C_{10}$ | $C_{11}$ | $C_{12}$ | $R$ |
| --- | --- | --- | --- | --- | --- | --- | --- | --- | --- | --- | --- | --- | --- |
| $C_{1}$ | {$s_{0}$} | {$s_{1}$,$s_{2}$,$s_{3}$} | {$s_{0}$} | {$s_{0}$} | {$s_{1}$,$s_{2}$} | {$s_{0}$} | {$s_{0}$} | {$s_{0}$} | {$s_{0}$} | {$s_{0}$} | {$s_{0}$} | {$s_{0}$} | {$s_{4}$,$s_{5}$} |
| $C_{2}$ | {$s_{0}$} | {$s_{0}$} | {$s_{0}$} | {$s_{0}$} | {$s_{3}$,$s_{4}$,$s_{5}$} | {$s_{0}$} | {$s_{1}$} | {$s_{0}$} | {$s_{0}$} | {$s_{0}$} | {$s_{0}$} | {$s_{0}$} | {$s_{3}$,$s_{4}$,$s_{5}$} |
| $C_{3}$ | {$s_{1}$,$s_{2}$,$s_{3}$} | {$s_{2}$,$s_{3}$,$s_{4}$} | {$s_{0}$} | {$s_{0}$} | {$s_{4}$,$s_{5}$} | {$s_{0}$} | {$s_{0}$} | {$s_{0}$} | {$s_{0}$} | {$s_{0}$} | {$s_{0}$} | {$s_{0}$} | {$s_{3}$,$s_{4}$,$s_{5}$} |
| $C_{4}$ | {$s_{0}$} | {$s_{-4}$,$s_{-3}$,$s_{-2}$} | {$s_{0}$} | {$s_{0}$} | {$s_{-2}$,$s_{-1}$} | {$s_{0}$} | {$s_{0}$} | {$s_{0}$} | {$s_{0}$} | {$s_{0}$} | {$s_{0}$} | {$s_{0}$} | {$s_{-5}$,$s_{-4}$} |
| $C_{5}$ | {$s_{3}$,$s_{4}$,$s_{5}$} | {$s_{4}$,$s_{5}$} | {$s_{1}$,$s_{2}$,$s_{3}$} | {$s_{3}$,$s_{4}$,$s_{5}$} | {$s_{0}$} | {$s_{3}$,$s_{4}$,$s_{5}$} | {$s_{4}$,$s_{5}$} | {$s_{0}$} | {$s_{0}$} | {$s_{0}$} | {$s_{0}$} | {$s_{0}$} | {$s_{4}$,$s_{5}$} |
| $C_{6}$ | {$s_{0}$} | {$s_{0}$} | {$s_{0}$} | {$s_{0}$} | {$s_{0}$} | {$s_{0}$} | {$s_{3}$,$s_{4}$,$s_{5}$} | {$s_{0}$} | {$s_{0}$} | {$s_{0}$} | {$s_{0}$} | {$s_{0}$} | {$s_{1}$,$s_{2}$} |
| $C_{7}$ | {$s_{1}$,$s_{2}$,$s_{3}$} | {$s_{0}$} | {$s_{1}$,$s_{2}$,$s_{4}$} | {$s_{2}$,$s_{3}$,$s_{5}$} | {$s_{0}$} | {$s_{0}$} | {$s_{0}$} | {$s_{0}$} | {$s_{0}$} | {$s_{0}$} | {$s_{0}$} | {$s_{0}$} | {$s_{1}$,$s_{2}$} |
| $C_{8}$ | {$s_{-2}$,$s_{-1}$,$s_{2}$} | {$s_{0}$} | {$s_{0}$} | {$s_{0}$} | {$s_{0}$} | {$s_{0}$} | {$s_{1}$,$s_{2}$} | {$s_{0}$} | {$s_{0}$} | {$s_{0}$} | {$s_{0}$} | {$s_{0}$} | {$s_{1}$,$s_{2}$} |
| $C_{9}$ | {$s_{1}$,$s_{2}$} | {$s_{0}$} | {$s_{0}$} | {$s_{4}$,$s_{5}$} | {$s_{0}$} | {$s_{1}$,$s_{2}$,$s_{5}$} | {$s_{0}$} | {$s_{3}$,$s_{4}$,$s_{5}$} | {$s_{0}$} | {$s_{0}$} | {$s_{0}$} | {$s_{0}$} | {$s_{3}$,$s_{4}$,$s_{5}$} |
| $C_{10}$ | {$s_{2}$,$s_{3}$,$s_{5}$} | {$s_{0}$} | {$s_{1}$,$s_{2}$,$s_{4}$} | {$s_{1}$,$s_{2}$} | {$s_{0}$,$s_{2}$,$s_{4}$} | {$s_{1}$,$s_{3}$} | {$s_{2}$,$s_{3}$} | {$s_{3}$,$s_{4}$} | {$s_{1}$,$s_{4}$,$s_{5}$} | {$s_{0}$(0)} | {$s_{0}$(0)} | {$s_{4}$,$s_{5}$} | {$s_{3}$,$s_{4}$} |
| $C_{11}$ | {$s_{0}$} | {$s_{0}$} | {$s_{2}$,$s_{3}$,$s_{5}$} | {$s_{0}$} | {$s_{0}$} | {$s_{0}$} | {$s_{0}$} | {$s_{0}$} | {$s_{0}$} | {$s_{0}$} | {$s_{0}$} | {$s_{0}$} | {$s_{1}$,$s_{3}$,$s_{4}$} |
| $C_{12}$ | {$s_{3}$,$s_{4}$,$s_{5}$} | {$s_{1}$,$s_{3}$,$s_{4}$} | {$s_{0}$} | {$s_{0}$} | {$s_{4}$,$s_{5}$} | {$s_{0}$} | {$s_{0}$} | {$s_{0}$} | {$s_{0}$} | {$s_{0}$} | {$s_{0}$} | {$s_{0}$} | {$s_{1}$,$s_{4}$,$s_{5}$} |
| $R$ | {$s_{0}$} | {$s_{2}$,$s_{3}$,$s_{4}$} | {$s_{2}$,$s_{3}$} | {$s_{-2}$,$s_{1}$,$s_{2}$} | {$s_{2}$,$s_{3}$,$s_{5}$} | {$s_{0}$} | {$s_{0}$} | {$s_{0}$} | {$s_{0}$} | {$s_{0}$} | {$s_{0}$} | {$s_{0}$} | {$s_{0}$} |

**Table S3** Strength of association between cause factors of FCM model

| $\omega_{ij}$ | $C_{1}$ | $C_{2}$ | $C_{3}$ | $C_{4}$ | $C_{5}$ | $C_{6}$ | $C_{7}$ | $C_{8}$ | $C_{9}$ | $C_{10}$ | $C_{11}$ | $C_{12}$ | $R$ |
| --- | --- | --- | --- | --- | --- | --- | --- | --- | --- | --- | --- | --- | --- |
| $C_{1}$ | 0 | 0.67 | 0 | 0 | 0.56 | 0 | 0 | 0 | 0 | 0 | 0 | 0 | 0.96 |
| $C_{2}$ | 0 | 0 | 0 | 0 | 0.91 | 0 | 0.6 | 0 | 0 | 0 | 0 | 0 | 0.81 |
| $C_{3}$ | 0.66 | 0.71 | 0 | 0 | 0.82 | 0 | 0 | 0 | 0 | 0 | 0 | 0 | 0.82 |
| $C_{4}$ | 0 | 0.17 | 0 | 0 | 0.32 | 0 | 0 | 0 | 0 | 0 | 0 | 0 | 0.07 |
| $C_{5}$ | 0.8 | 0.88 | 0.68 | 0.92 | 0 | 0.87 | 0.95 | 0 | 0 | 0 | 0 | 0 | 0.86 |
| $C_{6}$ | 0 | 0 | 0 | 0 | 0 | 0 | 0.91 | 0 | 0 | 0 | 0 | 0 | 0.68 |
| $C_{7}$ | 0.66 | 0 | 0.68 | 0.69 | 0 | 0 | 0 | 0 | 0 | 0 | 0 | 0 | 0.59 |
| $C_{8}$ | 0.38 | 0 | 0 | 0 | 0 | 0 | 0.65 | 0 | 0 | 0 | 0 | 0 | 0.58 |
| $C_{9}$ | 0.53 | 0 | 0 | 0.97 | 0 | 0.69 | 0 | 0.9 | 0 | 0 | 0 | 0 | 0.87 |
| $C_{10}$ | 0.81 | 0 | 0.66 | 0.27 | 0.76 | 0.64 | 0.61 | 0.79 | 0.87 | 0 | 0 | 0.95 | 0.77 |
| $C_{11}$ | 0 | 0 | 0.23 | 0 | 0 | 0 | 0 | 0 | 0 | 0 | 0 | 0 | 0.17 |
| $C_{12}$ | 0.87 | 0.83 | 0 | 0 | 0.95 | 0 | 0 | 0 | 0 | 0 | 0 | 0 | 0.92 |
| $R$ | 0 | 0.8 | 0.61 | 0.56 | 0.79 | 0 | 0 | 0 | 0 | 0 | 0 | 0 | 0 |
