# Supplementary material for: Probabilistic linguistic fuzzy cognitive maps: applications to the critical factors affecting the health of rural older adults
Source: BMC Med Inform Decis Mak. 2022 Nov 17;22:299. doi: 10.1186/s12911-022-02028-9 (PMC9673458; doi:10.1186/s12911-022-02028-9)
Supplement: Supplementary file 2 — Additional file 2. The codes of this article. [file 12911_2022_2028_MOESM2_ESM.docx]

Code 1

% The result is obtained by Z(t)=(1-t)/(1+t) and lammda = 1

%% The original causal relationship data of PLTS

% Step 1: Record initial information.

L={[0,0,0],[1,2,3],[0,0,0],[0,0,0],[1,2,2],[0,0,0],[0,0,0],[0,0,0],[0,0,0],[0,0,0],[0,0,0],[0,0,0],[3,5,5];

[0,0,0],[0,0,0],[0,0,0],[0,0,0],[3,4,5],[0,0,0],[1,1,1],[0,0,0],[0,0,0],[0,0,0],[0,0,0],[0,0,0],[2,4,5];

[1,2,3],[2,3,4],[0,0,0],[0,0,0],[2,5,5],[0,0,0],[0,0,0],[0,0,0],[0,0,0],[0,0,0],[0,0,0],[0,0,0],[2,4,5];

[0,0,0],[-4,-3,-2],[0,0,0],[0,0,0],[-2,-1,-1],[0,0,0],[0,0,0],[0,0,0],[0,0,0],[0,0,0],[0,0,0],[0,0,0],[-5,-4,-4];

[3,4,5],[4,5,5],[1,2,3],[3,4,5],[0,0,0],[3,4,5],[4,5,5],[0,0,0],[0,0,0],[0,0,0],[0,0,0],[0,0,0],[4,5,5];

[0,0,0],[0,0,0],[0,0,0],[0,0,0],[0,0,0],[0,0,0],[3,4,5],[0,0,0],[0,0,0],[0,0,0],[0,0,0],[0,0,0],[1,2,2];

[1,2,3],[0,0,0],[1,2,4],[2,3,5],[0,0,0],[0,0,0],[0,0,0],[0,0,0],[0,0,0],[0,0,0],[0,0,0],[0,0,0],[1,2,2];

[-2,-1,2],[0,0,0],[0,0,0],[0,0,0],[0,0,0],[0,0,0],[1,2,2],[0,0,0],[0,0,0],[0,0,0],[0,0,0],[0,0,0],[1,2,2];

[1,2,2],[0,0,0],[0,0,0],[4,5,5],[0,0,0],[1,2,5],[0,0,0],[2,4,5],[0,0,0],[0,0,0],[0,0,0],[0,0,0],[3,4,5];

[2,3,5],[0,0,0],[1,2,4],[-1,-2,-2],[0,2,4],[1,3,3],[2,3,3],[3,4,4],[1,4,5],[0,0,0],[0,0,0],[4,5,5],[3,4,4];

[0,0,0],[0,0,0],[-2,-3,-5],[0,0,0],[0,0,0],[0,0,0],[0,0,0],[0,0,0],[0,0,0],[0,0,0],[0,0,0],[0,0,0],[-1,-3,-4];

[3,4,5],[1,3,4],[0,0,0],[0,0,0],[4,5,5],[0,0,0],[0,0,0],[0,0,0],[0,0,0],[0,0,0],[0,0,0],[0,0,0],[1,4,5];

[0,0,0],[2,3,4],[2,3,3],[-2,1,2],[2,3,5],[0,0,0],[0,0,0],[0,0,0],[0,0,0],[0,0,0],[0,0,0],[0,0,0],[0,0,0];}; % The linguistic information of PLTS

P={[0,0,0],[0.5,0.3,0.2],[0,0,0],[0,0,0],[0.7,0.2,0],[0,0,0],[0,0,0],[0,0,0],[0,0,0],[0,0,0],[0,0,0],[0,0,0],[0.2,0.8,0];

[0,0,0],[0,0,0],[0,0,0],[0,0,0],[0.2,0.5,0.3],[0,0,0],[1,0,0],[0,0,0],[0,0,0],[0,0,0],[0,0,0],[0,0,0],[0.5,0.4,0.1];

[0.5,0.4,0.1],[0.3,0.4,0.2],[0,0,0],[0,0,0],[0.6,0.4,0],[0,0,0],[0,0,0],[0,0,0],[0,0,0],[0,0,0],[0,0,0],[0,0,0],[0.5,0.3,0.2];

[0,0,0],[0.5,0.3,0.2],[0,0,0],[0,0,0],[0.4,0.5,0],[0,0,0],[0,0,0],[0,0,0],[0,0,0],[0,0,0],[0,0,0],[0,0,0],[0.3,0.7,0];

[0.2,0.6,0.1],[0.2,0.7,0],[0.3,0.6,0.1],[0.2,0.4,0.4],[0,0,0],[0.4,0.5,0.1],[0.5,0.5,0],[0,0,0],[0,0,0],[0,0,0],[0,0,0],[0,0,0],[0.4,0.5,0];

[0,0,0],[0,0,0],[0,0,0],[0,0,0],[0,0,0],[0,0,0],[0.2,0.5,0.3],[0,0,0],[0,0,0],[0,0,0],[0,0,0],[0,0,0],[0.2,0.8,0];

[0.5,0.4,0.1],[0,0,0],[0.4,0.5,0.1],[0.5,0.3,0.1],[0,0,0],[0,0,0],[0,0,0],[0,0,0],[0,0,0],[0,0,0],[0,0,0],[0,0,0],[0.4,0.5,0];

[0.5,0.4,0.1],[0,0,0],[0,0,0],[0,0,0],[0,0,0],[0,0,0],[0.5,0.5,0],[0,0,0],[0,0,0],[0,0,0],[0,0,0],[0,0,0],[0.5,0.4,0];

[0.3,0.5,0],[0,0,0],[0,0,0],[0.3,0.7,0],[0,0,0],[0.4,0.5,0.1],[0,0,0],[0.2,0.4,0.4],[0,0,0],[0,0,0],[0,0,0],[0,0,0],[0.5,0.3,0.2];

[0.3,0.5,0.2],[0,0,0],[0.6,0.3,0.1],[0.3,0.5,0],[0.2,0.3,0.5],[0.4,0.5,0],[0.3,0.5,0],[0.2,0.7,0],[0.2,0.5,0.3],[0,0,0],[0,0,0],[0.5,0.5,0],[0.4,0.5,0];

[0,0,0],[0,0,0],[0.5,0.4,0.1],[0,0,0],[0,0,0],[0,0,0],[0,0,0],[0,0,0],[0,0,0],[0,0,0],[0,0,0],[0,0,0],[0.1,0.4,0.5];

[0.4,0.5,0.1],[0.1,0.4,0.5],[0,0,0],[0,0,0],[0.5,0.5,0],[0,0,0],[0,0,0],[0,0,0],[0,0,0],[0,0,0],[0,0,0],[0,0,0],[0.1,0.4,0.5];

[0,0,0],[0.3,0.4,0.3],[0.3,0.5,0],[0.3,0.2,0.5],[0.3,0.6,0.1],[0,0,0],[0,0,0],[0,0,0],[0,0,0],[0,0,0],[0,0,0],[0,0,0],[0,0,0];}; % The probability of LTs

% Step 2£ºNormalize the probabilities of LTs

[m,n]=size(P);

for i=1:m

for j=1:n

if sum(P{i,j})~=0

NP{i,j}=P{i,j}./sum(P{i,j});

else

NP{i,j}=P{i,j};

end

end

end

% Step 3£ºTransform the LTs into Hesitant fuzzy element

tao=5;

[p,q]=size(L);

for k=1:p

for h=1:q

% for j=1:length(L{p})

% if L{k,h}(j)>0 & L{k,h}(j)==0

HFE{k,h}=L{k,h}./(2*tao)+1/2;

end

end

%% Step 4 Iterative Process -- PL-FCM calculation process

% Iterative program 1

% Illustration of Symbols£ºC_0£ºinitial status£»H:Transformation Matrix£»lambda: the parameter of hyperbolic tangents£»

C_0_L={[-3,0,3],[-3,0,3],[-3,0,3],[-3,0,3],[-3,0,3],[-3,0,3],[-3,0,3],[-3,0,3],[-3,0,3],[-3,0,3],[-3,0,3],[-3,0,3],[-3,0,3]};

C_0_P={[0.15,0.2,0.65],[0.1,0.3,0.6],[0.75,0,0.25],[0.3,0,0.7],[0.15,0,0.85],[0.4,0,0.6],[0.3,0,0.7],[0.4,0.55,0.05],[0.1,0.75,0.15],[0.6,0.35,0.05],[0.3,0.5,0.2],[0.1,0.4,0.5],[0.15,0.4,0.45]};

H=HFE';

P=NP';

f=@(x,lambda) (exp(lambda.*x)-exp(-lambda.*x))./(exp(lambda.*x)+exp(-lambda.*x)); % hyperbolic tangents

lambda=1; % By setting different lambda, it is possible to obtain different sorting of results in Comparative Analysis.

C_1_L=cell(1,13);

C_1_P=cell(1,13);

for k=1:length(C_0_L)

C_0_L{k}=C_0_L{k}./(2*tao)+1/2;

end

C_00_P=C_0_P;

for i=1:13

for j=1:13

if j==i

continue

end

C_00_L{j}=1-C_0_L{j}.*H{j,i};

C_00_P{j}=1-C_0_P{j}.*P{j,i};

end

C_00_L{i}=1-C_0_L{i};

C_00_P{i}=1-C_0_P{i};

original_L=ones(1,3);

original_P=ones(1,3);

for k=1:13

original_L=original_L.*C_00_L{k};

original_P=original_P.*C_00_P{k};

end

x1_L=ones(1,3)-original_L;

x1_P=ones(1,3)-original_P;

C_1_L{i}=f(x1_L,lambda);

C_1_P{i}=f(x1_P,lambda);

end

for i=1:length(C_1_P)

C_1_P{i}=C_1_P{i}./sum(C_1_P{i});

end

%% Iterate program 2, when the number of iterations is not required, you can keep clicking "run section" botton until the result is stable or less than a certain threshold.

C_0_L=C_1_L;

C_0_P=C_1_P;

C_00_L=C_0_L;

C_00_P=C_0_P;

for i=1:13

for j=1:13

if j==i

continue

end

C_00_L{j}=(1-C_0_L{j}.*H{j,i});

C_00_P{j}=(1-C_0_P{j}.*P{j,i});

end

C_00_L{i}=1-C_0_L{i};

C_00_P{i}=1-C_0_P{i};

original_L=ones(1,3);

original_P=ones(1,3);

for k=1:13

original_L=original_L.*C_00_L{k};

original_P=original_P.*C_00_P{k};

end

x1_L=ones(1,3)-original_L;

x1_P=ones(1,3)-original_P;

C_1_L{i}=f(x1_L,lambda);

C_1_P{i}=f(x1_P,lambda);

end

for i=1:length(C_1_P)

C_1_P{i}=C_1_P{i}./sum(C_1_P{i});

end

%% Step 5£ºCalculate the correlation between factors and results.

for i=1:13

L{1,i}=(C_1_L{1,i}-1/2)*2*tao;

end

for j=1:13

s{1,j}=L{1,j}.*C_1_P{1,j};

end

for k=1:12

d(k)=sqrt(sum((s{k}-s{13}).^2))./3;

end

% Remark: By using different correlation functions, we can obtain different results, which are shown in the comparative analysis.

% Z1=@(t) 1-t;

Z2=@(t) (1-t)./(1+t);

% Z3=@(t) 1-t.*exp(t-1);

% Z4=@(t) 1-t.^2;

% x1=(Z1(d)-Z1(1))/(Z1(0)-Z1(1));

x2=(Z2(d)-Z2(1))/(Z2(0)-Z2(1));

% x3=(Z3(d)-Z3(1))/(Z3(0)-Z3(1));

% x4=(Z4(d)-Z4(1))/(Z4(0)-Z4(1));

%% Step 6: order the final results.

% [number1,value1]=sort(x1);

[number2,value2]=sort(x2);

% [number3,value3]=sort(x3);

% [number4,value4]=sort(x4);

Code 2

%% The comparation analysis between HFLCM and PLFCM

% Step 1£ºThe original causal relationship data of HFLTS

clear; clc;

L={[0,0,0],[1,2,3],[0,0,0],[0,0,0],[1,2,2],[0,0,0],[0,0,0],[0,0,0],[0,0,0],[0,0,0],[0,0,0],[0,0,0],[3,5,5];

[0,0,0],[0,0,0],[0,0,0],[0,0,0],[3,4,5],[0,0,0],[1,1,1],[0,0,0],[0,0,0],[0,0,0],[0,0,0],[0,0,0],[2,4,5];

[1,2,3],[2,3,4],[0,0,0],[0,0,0],[2,5,5],[0,0,0],[0,0,0],[0,0,0],[0,0,0],[0,0,0],[0,0,0],[0,0,0],[2,4,5];

[0,0,0],[-4,-3,-2],[0,0,0],[0,0,0],[-2,-1,-1],[0,0,0],[0,0,0],[0,0,0],[0,0,0],[0,0,0],[0,0,0],[0,0,0],[-5,-4,-4];

[3,4,5],[4,5,5],[1,2,3],[3,4,5],[0,0,0],[3,4,5],[4,5,5],[0,0,0],[0,0,0],[0,0,0],[0,0,0],[0,0,0],[4,5,5];

[0,0,0],[0,0,0],[0,0,0],[0,0,0],[0,0,0],[0,0,0],[3,4,5],[0,0,0],[0,0,0],[0,0,0],[0,0,0],[0,0,0],[1,2,2];

[1,2,3],[0,0,0],[1,2,4],[2,3,5],[0,0,0],[0,0,0],[0,0,0],[0,0,0],[0,0,0],[0,0,0],[0,0,0],[0,0,0],[1,2,2];

[-2,-1,2],[0,0,0],[0,0,0],[0,0,0],[0,0,0],[0,0,0],[1,2,2],[0,0,0],[0,0,0],[0,0,0],[0,0,0],[0,0,0],[1,2,2];

[1,2,2],[0,0,0],[0,0,0],[4,5,5],[0,0,0],[1,2,5],[0,0,0],[2,4,5],[0,0,0],[0,0,0],[0,0,0],[0,0,0],[3,4,5];

[2,3,5],[0,0,0],[1,2,4],[-1,-2,-2],[0,2,4],[1,3,3],[2,3,3],[3,4,4],[1,4,5],[0,0,0],[0,0,0],[4,5,5],[3,4,4];

[0,0,0],[0,0,0],[-2,-3,-5],[0,0,0],[0,0,0],[0,0,0],[0,0,0],[0,0,0],[0,0,0],[0,0,0],[0,0,0],[0,0,0],[-1,-3,-4];

[3,4,5],[1,3,4],[0,0,0],[0,0,0],[4,5,5],[0,0,0],[0,0,0],[0,0,0],[0,0,0],[0,0,0],[0,0,0],[0,0,0],[1,4,5];

[0,0,0],[2,3,4],[2,3,3],[-2,1,2],[2,3,5],[0,0,0],[0,0,0],[0,0,0],[0,0,0],[0,0,0],[0,0,0],[0,0,0],[0,0,0];};

% Step 2£ºTransforme Linguistic terms information into Hesitant fuzzy element

tao=5;

[p,q]=size(L);

for k=1:p

for h=1:q

HFE{k,h}=L{k,h}./(2*tao)+1/2;

end

end

%% Step 3£ºIteration Process-HFLCM

% Iterative program 1

% Illustration of Symbols£ºC_0£ºinitial status£»H:Transformation Matrix£»lambda: the parameter of hyperbolic tangents£»

C_0_L={[0,1],[0,1],[0,1],[0,1],[0,1],[0,1],[0,1],[0,1],[0,1],[0,1],[0,1],[0,1],[0,1],[0,1],[0,1]};

C_0_L={[-3,0,3],[-3,0,3],[-3,0,3],[-3,0,3],[-3,0,3],[-3,0,3],[-3,0,3],[-3,0,3],[-3,0,3],[-3,0,3],[-3,0,3],[-3,0,3],[-3,0,3]};

H=HFE';

f=@(x,lambda) (exp(lambda.*x)-exp(-lambda.*x))./(exp(lambda.*x)+exp(-lambda.*x));

lambda=1;

C_1_L=cell(1,13);

for k=1:length(C_0_L);

C_0_L{k}=C_0_L{k}./(2*tao)+1/2;

end

for i=1:13

for j=1:13

if j==i

continue

end

C_00_L{j}=1-C_0_L{j}.*H{j,i};

end

C_00_L{i}=1-C_0_L{i};

original_L=ones(1,3);

for k=1:13

original_L=original_L.*C_00_L{k};

end

x1_L=ones(1,3)-original_L;

C_1_L{i}=f(x1_L,lambda);

end

%% Iterate program 2, when the number of iterations is not required, you can keep clicking "run section" botton until the result is stable or less than a certain threshold.

C_0_L=C_1_L;

C_00_L=C_0_L;

for i=1:13

for j=1:13

if j==i

continue

end

C_00_L{j}=(1-C_0_L{j}.*H{j,i});

end

C_00_L{i}=1-C_0_L{i};

original_L=ones(1,3);

for k=1:13

original_L=original_L.*C_00_L{k};

end

x1_L=ones(1,3)-original_L;

lambda=1;

C_1_L{i}=f(x1_L,lambda);

end

%% Step 4£ºCalculate the correlation between factors and results.

for i=1:13

L{1,i}=(C_1_L{1,i}-1/2)*2*tao;

end

for j=1:13

s{1,j}=L{1,j};

end

for k=1:12

d(k)=sqrt(sum((s{k}-s{13}).^2))./3;

end

% Remark: By using different correlation functions, we can obtain different

% results, which are shown in the comparative analysis. However, in this

% part, we select Z2 as the similarity measures.

Z1=@(t) 1-t;

Z2=@(t) (1-t)./(1+t);

Z3=@(t) 1-t.*exp(t-1);

Z4=@(t) 1-t.^2;

x1=(Z1(d)-Z1(1))/(Z1(0)-Z1(1));

x2=(Z2(d)-Z2(1))/(Z2(0)-Z2(1));

x3=(Z3(d)-Z3(1))/(Z3(0)-Z3(1));

x4=(Z4(d)-Z4(1))/(Z4(0)-Z4(1));

%% Step 5: order the final results.

[number1,value1]=sort(x1);

[number2,value2]=sort(x2);

[number3,value3]=sort(x3);

[number4,value4]=sort(x4);
